# Delayed surgery for more than 9 weeks induces worse survival outcomes in locally advanced rectal cancer patients with poor response to neoadjuvant chemoradiotherapy: a propensity score matched cohort study

**DOI:** 10.1093/gastro/goaf060

**Published:** 2025-07-02

**Authors:** Hao Wang, Yuan Li, Xinyu Ge, Shaopu Lian, Cheng Feng, Weili Zhang, E-Er-Man-Bie-Ke Jin-Si-Han, Long Yu, Qingjian Ou, Peirong Ding, Zhizhong Pan, Zhenhai Lu

**Affiliations:** Department of Colorectal Surgery, State Key Laboratory of Oncology in South China, Guangdong Provincial Clinical Research Center for Cancer, Sun Yat-sen University Cancer Center, Guangzhou, Guangdong, P. R. China; Department of Colorectal Surgery, State Key Laboratory of Oncology in South China, Guangdong Provincial Clinical Research Center for Cancer, Sun Yat-sen University Cancer Center, Guangzhou, Guangdong, P. R. China; Department of Cancer Prevention Center, State Key Laboratory of Oncology in South China, Guangdong Provincial Clinical Research Center for Cancer, Sun Yat-sen University Cancer Center, Guangdong, P. R. China; Department of Colorectal Surgery, State Key Laboratory of Oncology in South China, Guangdong Provincial Clinical Research Center for Cancer, Sun Yat-sen University Cancer Center, Guangzhou, Guangdong, P. R. China; Department of Thyroid & Galactophore Surgery, People's Hospital of Longhua, Shenzhen, Guangdong, P. R. China; Department of Colorectal Surgery, State Key Laboratory of Oncology in South China, Guangdong Provincial Clinical Research Center for Cancer, Sun Yat-sen University Cancer Center, Guangzhou, Guangdong, P. R. China; Department of Colorectal Surgery, State Key Laboratory of Oncology in South China, Guangdong Provincial Clinical Research Center for Cancer, Sun Yat-sen University Cancer Center, Guangzhou, Guangdong, P. R. China; Department of Colorectal Surgery, State Key Laboratory of Oncology in South China, Guangdong Provincial Clinical Research Center for Cancer, Sun Yat-sen University Cancer Center, Guangzhou, Guangdong, P. R. China; Department of Colorectal Surgery, State Key Laboratory of Oncology in South China, Guangdong Provincial Clinical Research Center for Cancer, Sun Yat-sen University Cancer Center, Guangzhou, Guangdong, P. R. China; Department of Colorectal Surgery, State Key Laboratory of Oncology in South China, Guangdong Provincial Clinical Research Center for Cancer, Sun Yat-sen University Cancer Center, Guangzhou, Guangdong, P. R. China; Department of Colorectal Surgery, State Key Laboratory of Oncology in South China, Guangdong Provincial Clinical Research Center for Cancer, Sun Yat-sen University Cancer Center, Guangzhou, Guangdong, P. R. China; Department of Colorectal Surgery, State Key Laboratory of Oncology in South China, Guangdong Provincial Clinical Research Center for Cancer, Sun Yat-sen University Cancer Center, Guangzhou, Guangdong, P. R. China

**Keywords:** neoadjuvant chemoradiotherapy, locally advanced rectal cancer, tumor response, waiting interval, surgery

## Abstract

**Background:**

The association between delayed surgery and survival outcomes in locally advanced rectal cancer patients with a poor response to neoadjuvant chemoradiotherapy (nCRT) remains unclear. This study aimed to determine the optimal timing of surgery following nCRT in these patients and to explore the association between delayed surgery and survival outcomes.

**Methods:**

Restricted cubic spline curves were used to determine the optimal timing of surgery for patients with a poor response to nCRT (ypT2–4N0 or ypTxN+). The patients were divided into two groups: the early surgery group and the delayed surgery group. Propensity score matching (PSM) analysis was employed to reduce the selection bias and survival analysis was conducted to assess the survival differences. Immunostaining of post-operative specimens was performed to investigate whether the difference in survival was associated with the CD8^+^ T-cell density in the tumor.

**Results:**

A total of 583 patients were enrolled in this study. The optimal timing for surgery was determined to be 9 weeks after nCRT. In PSM analysis, delayed surgery was associated with worse disease-free survival (63.0% vs 76.3% at 5 years, 53.0% vs 76.3% at 10 years; *P *=* *0.003) and cancer-specific survival (72.9% vs 85.5% at 5 years, 60.1% vs 81.8% at 10 years; *P *=* *0.001). Immunostaining analysis showed that longer waiting times were associated with decreased CD8^+^ T-cell density in tumors (*P *=* *0.017).

**Conclusions:**

Patients who had a poor tumor response after nCRT, detected by using magnetic resonance imaging restaging or other assessments, need timely radical surgery without delay.

## Introduction

Neoadjuvant chemoradiotherapy (nCRT) followed by radical surgery is the established standard treatment for locally advanced rectal cancer (LARC) [1, 2]. Approximately 15%–20% of LARC patients can achieve pathological complete response (pCR) after nCRT; these patients had a lower local recurrence rate (LR) and higher disease-free survival (DFS) and overall survival (OS) rates than non-pCR patients [3–5]. Therefore, maximizing the pCR rate has become one of the current focuses in the clinical management of LARC patients.

The interval between nCRT and surgery has been identified as a significant predictor for improving tumor response after nCRT, thereby improving pCR rates [6–10]. The Lyon R90-01 trial was the first to investigate the correlation between the interval and the pCR rate, suggesting that delaying surgery for 6–8 weeks after nCRT can improve the pCR rate [6]. Recently, the optimal interval between nCRT and surgery has further increased. A meta-analysis indicated that postponing surgery until the 10th week after the completion of nCRT would be the optimal time to achieve pCR [10], considering that the prolonged interval did not affect survival outcomes. For patients who eventually achieve a pCR or near pCR after nCRT, the decision related to this practice is evidently beneficial. However, a large portion of patients who received neoadjuvant treatment failed to gain significant tumor regression. The impact of delayed surgery on survival outcomes in these patients remains unknown. Therefore, it is necessary to determine the optimal timing for delaying surgery in those poor responders.

In recent years, there has been a significant increase in research regarding the role of tumor-infiltrating lymphocytes. Many studies have demonstrated that elevated levels of CD3^+^ or CD8^+^ T cells in the tumor microenvironment, particularly CD8^+^ T cells, are associated with improved survival outcomes [11–14]. Investigating the relationship between neoadjuvant treatment and intratumoral lymphocyte infiltration may yield valuable insights into the prognosis of rectal cancer.

This study aimed to determine the optimal timing of surgery in LARC patients who had a poor response to nCRT and to examine the potential impact of the waiting intervals on survival outcomes. Moreover, the study explored lymphocyte infiltration within the tumor to elucidate the possible mechanisms underlying the observed survival differences in patients with different waiting intervals.

## Patients and methods

### Study design and participants

This was a propensity score matched retrospective study. We included patients who were diagnosed with clinical Stage II or III rectal cancer from a large tertiary cancer center in China between January 2010 and July 2020. The study was approved by the Ethics Committee of Sun Yat-sen University Cancer Center (Guangzhou, China; approval number: SL-B2024-086–01) and informed consent to collect clinical data was obtained from each patient. The study was conducted in accordance with the Declaration of Helsinki. This study has been registered in the Research Registry (registration ID: researchregistry10242).

Patient demographics and treatment details were obtained from the electronic medical record system, while follow-up data were collected from a tracking system. The inclusion criteria were as follows: (i) rectal adenocarcinoma; (ii) receipt of long-course chemoradiotherapy; and (iii) those with a poor response to nCRT, defined as a pathological stage of ypT2–4N0 or ypN+. The exclusion criteria were as follows: (i) concurrent malignancies; (ii) receiving palliative surgery; or (iii) pathological stage of ypT0–1N0.

### Neoadjuvant chemoradiotherapy and surgery

All enrolled patients received preoperative long-course radiotherapy at a dose of 50 Gy/25 fractions. Some patients underwent capecitabine-based concurrent chemotherapy, administered at a dose of 825 mg/m^2^ twice daily for 5 days/week. A subset of patients, particularly those in the later stages of the study (since about 2014), received an intensified neoadjuvant chemotherapy based on the XELOX regimen (oxaliplatin 130 mg/m^2^ on Day 1; capecitabine 1,000 mg/m^2^ twice daily from Days 1 to 14), which included two cycles of XELOX with concurrent radiotherapy, along with one cycle of induction chemotherapy before radiotherapy and one cycle of consolidation chemotherapy after radiotherapy. The surgical interval was defined as the waiting time between the completion of radiotherapy and surgery. Surgery was recommended within 6–8 weeks after the completion of nCRT and total mesorectal excision was mandatory. All surgical procedures were performed by experienced colorectal surgeons. After surgery, adjuvant chemotherapy was recommended to complete a total of 6 months of perioperative systemic therapy.

### Tumor response

Tumor response was defined based on the pathological Tumor Node Metastasis stage (ypTNM) (the AJCC TNM 8th edition) [15]. Patients who achieved pCR (ypT0N0M0) or near pCR (ypT1N0M0) were considered to be responding well to nCRT. Conversely, patients with pathological stage ypT2–4N0M0 or ypTxN+M0 were considered as having a poor response to nCRT.

### Follow-up

Regular assessments, including clinical examination, tumor marker measurement, abdominal ultrasound, and chest radiography, were performed every 3 months for the first 3 years, then every 6 months until the fifth year, and annually thereafter. Additionally, enhanced computed tomography examinations of the chest, abdomen, and pelvis, as well as pelvic enhanced magnetic resonance imaging (MRI) and colonoscopy were performed on an annual basis. The date of final follow-up for this study was April 2022. The primary outcomes were DFS and cancer-specific survival (CSS). DFS was defined as the period between surgical resection and the occurrence of local or distant recurrence or death from rectal cancer. CSS was defined as the time between surgical resection and death caused by rectal cancer.

### Immunohistopathological analysis

We collected 42 surgical specimens for immunostaining. The surgical specimens were sectioned into 3-μm-thick slices. The tissue slices were incubated with CD8 primary antibody (Abcam, Cambridge, MA, USA) and then with secondary antibody. Whole-slide tissue array images of immunostaining were acquired by using a digital slide scanner (Jiangfeng Technology Development Co., Ltd, Shanghai, China). The boundaries of normal tissue, non-cancerous fibrotic areas after tumor regression, residual cancer cells, and clusters were manually delineated by using HALO software (Indica Labs, Corrales, NM, USA). The overall density of CD8^+^ lymphocytes (cells/mm^2^) within the global residual tumor area after neoadjuvant treatment was quantified by using HALO software.

### Statistical analysis

Categorical variables were assessed by using the chi-squared test or Fisher’s exact test. Continuous variables were evaluated by using the *t*-test or Mann–Whitney *U* test. Survival curves were generated by using the Kaplan–Meier method and the log-rank test was performed. The restricted mean survival time method was employed to facilitate a more comprehensive comparison of survival difference [16, 17]. The life-expectancy difference was also calculated. Cox proportional hazard regression model was utilized for both univariate and multivariate survival analyses. All variables that achieved a *P*-value of <0.05 in the univariate analysis were included in the multivariate analysis.

Restricted cubic spline curves were employed to continuously assess the association between the surgical interval and survival outcomes [18, 19]. In order to achieve an optimal fit to the main spline, the minimum number of knots, ranging from three to seven, was determined based on the Akaike Information Criterion [19]. Using the cut-off value derived from the restricted cubic spline curves, patients were divided into two groups: the early surgery group and the delayed surgery group.

To balance the potential confounding factors between the groups, propensity score matching (PSM) was employed. A logistic regression model was used to generate the propensity scores and one-to-one nearest-neighbor matching with a caliper of 0.1 was applied. Due to significant differences in the baseline characteristics and clinical significance, covariates including age, year of diagnosis, type of neoadjuvant chemotherapy regimen, and number of adjuvant chemotherapy cycles were included in the matching process.

Subgroup analysis was performed to assess the robustness of the results across various subgroups. Additionally, we conducted a sensitivity analysis to further evaluate the reliability of pathological staging compared with the tumor regression grading (TRG) system as a standard for determining the tumor response. In all analyses, a two-sided *P*-value of <0.05 was considered statistically significant. Statistical analyses were performed by using R software (version 4.2.1).

## Results

### Patient characteristics

**Figure 1.**
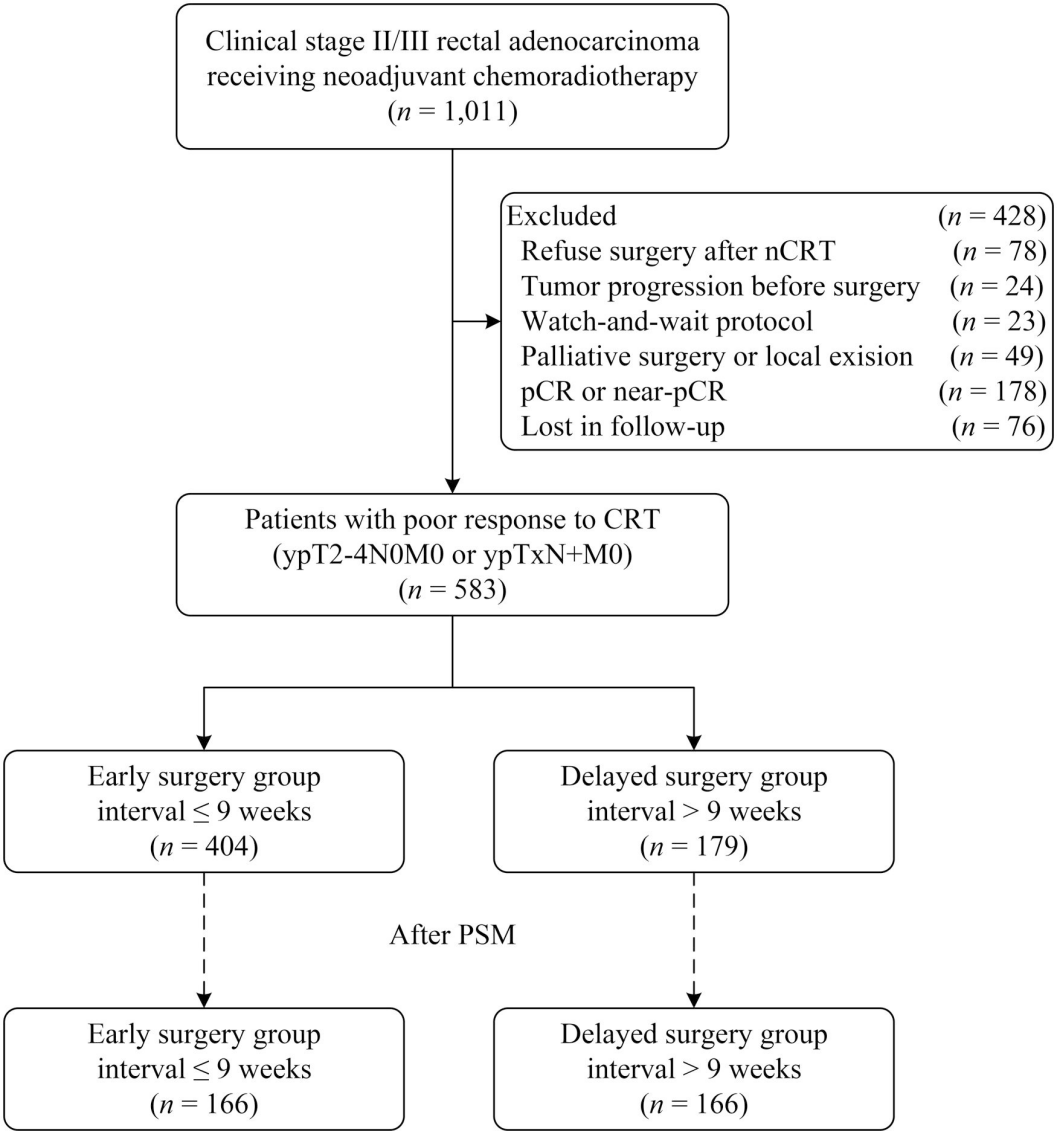
Study flow diagram.

Between January 2010 and July 2020, a total of 583 patients who had a poor pathological response to nCRT were included in this study (Figure 1). There were 373 (64.0%) men and 210 (36.0%) women, with a median age of 57 (interquartile range, 48–64) years. The median surgical interval was 57 (interquartile range, 50–66) days among all patients.

**Figure 2.**
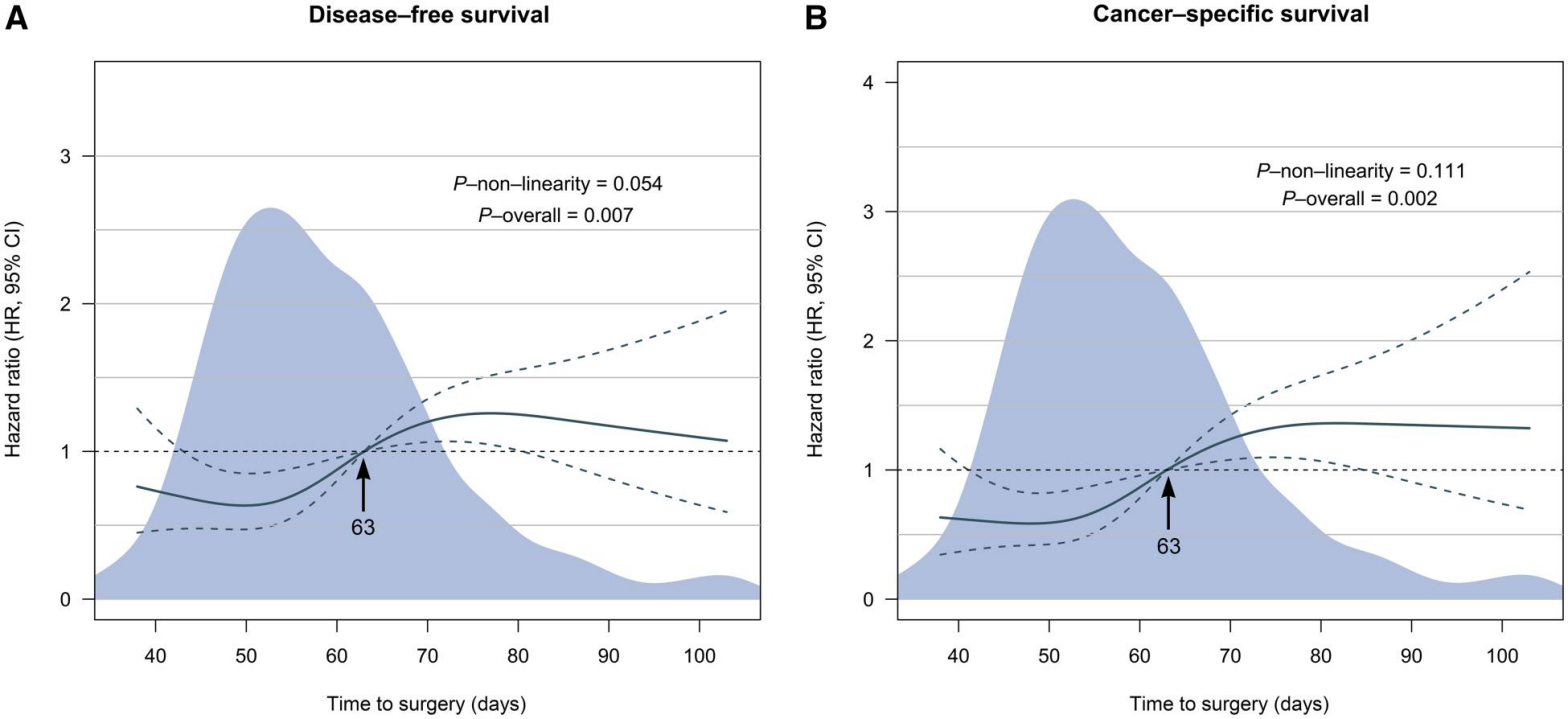
Association between the timing of surgery and (A) DFS and (B) CSS. HRs are indicated by solid lines and 95% CIs by dashed lines. All reference points are set at 63 days, with knots placed at the 5th, 35th, 65th, and 95th centiles.

Restricted cubic spline curves displayed a negative linear association between the waiting time and DFS and CSS (all *P for nonlinearity* > 0.05) (Figure 2). When the waiting time was ≤63 days, the hazard ratio (HR) was low, whereas it became high when the waiting time was >63 days. Therefore, the optimal cut-off value was determined to be 9 weeks. Accordingly, participants were divided into two groups: the early surgery group (≤9 weeks group), consisting of 404 patients, and the delayed surgery group (>9 weeks group), comprising 179 patients.

The two patient groups were balanced in pretreatment characteristics, except for the year of diagnosis (Supplementary Table S1). More patients received oxaliplatin-based nCRT in the early surgery group than in the delayed surgery group, but the number of XELOX cycles was similar. After surgery, a higher percentage of patients underwent adjuvant chemotherapy in the early surgery group than in the delayed surgery group. In addition, the duration of adjuvant chemotherapy was also different between the two groups. We performed one-to-one nearest-neighbor PSM, resulting in 166 patients in each cohort, with no significant differences between the early and delayed surgery groups (Table 1).

**Table 1.** Patient clinical and demographic characteristics after PSM

Variable	No. of patients (%)		*P*-value
	≤9 weeks (*n *=* *166)	>9 weeks (*n *=* *166)	
Age, median (IQR), years	57 (42–72)	57 (41–73)	0.569
Sex			>0.999
Male	104 (62.7)	104 (62.7)	
Female	62 (37.3)	62 (37.3)	
BMI, median (IQR), kg/m^2^	22.8 (17.4–28.8)	25.2 (20.2–31.1)	0.158
ECOG			0.807
0	118 (71.1)	121 (72.9)	
1	48 (28.9)	45 (27.1)	
Comorbidity			0.083
0	123 (74.1)	137 (82.5)	
≥1	43 (25.9)	29 (17.5)	
CEA, median (IQR), ng/mL	4.2 (2.1–7.1)	3.9 (2.5–8.2)	0.402
Distance from the anal verge, median (IQR), cm	6.0 (4.0–8.0)	6.0 (4.0–8.0)	0.718
Tumor location			0.479
Anterior	53 (31.9)	49 (29.5)	
Lateral	18 (10.8)	13 (7.8)	
Posterior	18 (10.8)	14 (8.4)	
Circumferential	77 (46.4)	90 (54.2)	
Depth of tumor invasion, median (IQR), mm	15.1 (11.6–18.2)	15.9 (11.4–18.8)	0.659
Longitudinal length of tumor, median (IQR), mm	49.5 (38.4–60.5)	50.0 (39.0–61.0)	0.790
MRF			0.096
Positive	75 (45.2)	56 (33.7)	
Negative	84 (50.6)	100 (60.2)	
Unknown	7 (4.2)	10 (6.0)	
EMVI			0.894
Positive	65 (39.2)	61 (36.7)	
Negative	91 (54.8)	94 (56.6)	
Unknown	10 (6.0)	11 (6.6)	
cT stage			0.603
2	5 (3.0)	5 (3.0)	
3	101 (60.8)	92 (55.4)	
4	60 (36.1)	69 (41.6)	
cN stage			0.406
0	28 (16.9)	31 (18.7)	
1	90 (54.2)	78 (47.0)	
2	48 (28.9)	57 (34.3)	
Histological grade			0.703
Moderate, moderate-high, and high grades	134 (80.7)	128 (77.1)	
Low and low-moderate grades	26 (15.7)	30 (18.1)	
Signet-ring and mucinous adenocarcinoma	5 (3.0)	7 (4.2)	
Unknown	1 (0.6)	1 (0.6)	
Neoadjuvant chemotherapy			>0.999
XELOX	96 (57.8)	96 (57.8)	
Capecitabine	70 (42.2)	70 (42.2)	
Cycles of XELOX			0.861
1	6 (3.6)	9 (5.4)	
2	20 (12.0)	22 (13.3)	
3	20 (12.0)	22 (13.3)	
4	50 (30.1)	43 (25.9)	
Grade 3/4 adverse events of chemotherapy^a^			
Any events	30 (18.1)	29 (17.5)	>0.999
Anemia	13 (7.8)	15 (9.0)	0.844
Leukopenia	11 (6.6)	9 (5.4)	0.818
Thrombocytopenia	9 (5.4)	8 (4.8)	>0.999
Nausea/vomiting	3 (1.8)	2 (1.2)	>0.999
Diarrhea	4 (2.4)	3 (1.8)	>0.999
Liver injury	0	0	>0.999
Renal injury	1 (0.6)	1 (0.6)	>0.999
Neurotoxicity	1 (0.6)	0	>0.999
Surgical type			0.110
Laparoscopic	98 (59.0)	113 (68.1)	
Open	68 (41.0)	53 (31.9)	
Surgery procedure			0.681
Anterior resection	128 (77.1)	125 (75.3)	
Abdominoperineal resection	37 (22.3)	38 (22.9)	
Hartmann	1 (0.6)	3 (1.8)	
Temporary or permanent stoma			0.313
Yes	71 (42.8)	61 (36.7)	
No	95 (57.2)	105 (63.3)	
ypT stage			0.975
0/1	4 (2.4)	3 (1.8)	
2	46 (27.7)	46 (27.7)	
3	102 (61.4)	101 (60.8)	
4	14 (8.4)	16 (9.6)	
ypN stage			>0.999
0	117 (70.5%)	117 (70.5%)	
1	39 (23.5%)	38 (22.9%)	
2	10 (6.0%)	11 (6.6%)	
Post-operative lymph node retrieval, medium (IQR)	8.0 (5.0–9.0)	8.0 (4.0–9.0)	0.721
Tumor deposit			0.849
Presence	16 (9.6%)	14 (8.4%)	
Absence	150 (90.4%)	152 (91.6%)	
Nerve invasion			>0.999
Presence	16 (9.6%)	15 (9.0%)	
Absence	150 (90.4%)	151 (91.0%)	
Vessel carcinoma embolus			0.194
Presence	15 (9.0%)	8 (4.8%)	
Absence	151 (91.0%)	158 (95.2%)	
MMR status			0.540
dMMR	4 (2.4%)	7 (4.2%)	
pMMR	127 (76.5%)	126 (75.9%)	
Unknown	35 (21.1%)	33 (19.9%)	
Surgical complications			
Any complication	22 (13.3)	21 (12.7)	>0.999
Anastomotic leakage	9 (5.4)	11 (6.6)	0.818
Anastomotic bleeding	4 (2.4)	4 (2.4)	>0.999
Abdominal infection	1 (0.6)	2 (1.2)	>0.999
Bowel obstruction	2 (1.2)	4 (2.4)	0.685
Wound infection	0 (0.0)	0 (0.0)	>0.999
Bleeding	2 (1.2)	1 (0.6)	>0.999
Dysuria	2 (1.2)	1 (0.6)	>0.999
Lymphatic leakage	2 (1.2)	1 (0.6)	>0.999
Clavien Dindo grade			>0.999
I	10 (6.0)	9 (5.4)	
II	10 (6.0)	9 (5.4)	
IIIa	1 (0.6)	2 (1.2)	
IIIb	1 (0.6)	1 (0.6)	
Adjuvant chemotherapy			0.687
No	33 (19.9)	37 (22.3)	
Yes	133 (80.1)	129 (77.7)	
Cycles of adjuvant chemotherapy			0.884
0	33 (19.9)	37 (22.3)	
1–3	39 (23.5)	38 (22.9)	
4–6	70 (42.2)	68 (41.0)	
Unknown	24 (14.5)	23 (13.9)	
Year of diagnosis			0.939
2010–2013	34 (20.5)	36 (21.7)	
2014–2016	68 (41.0)	69 (41.6)	
2017–2020	64 (38.6)	61 (36.7)	

a National Cancer Institute Common Terminology Criteria for Adverse Events version 4.0.

IQR = interquartile range, BMI = body mass index, ECOG = Eastern Cooperative Oncology Group, MRF = mesorectal fascia, EMVI = extramural vascular invasion, CEA = carcinoembryonic antigen, MMR = mismatch repair, dMMR = mismatch repair deficient, pMMR = mismatch repair proficient.

### Survival analysis

The median follow-up was 78 months. During the follow-up, patients in the delayed surgery group had significantly worse DFS and CSS than those in the early surgery group (*P *<* *0.05) (Supplementary Figure S1). Additionally, our data indicated that patients had a trend for lower survival rates as the waiting time increased, especially for >12 weeks (Supplementary Figure S2).

**Figure 3.**
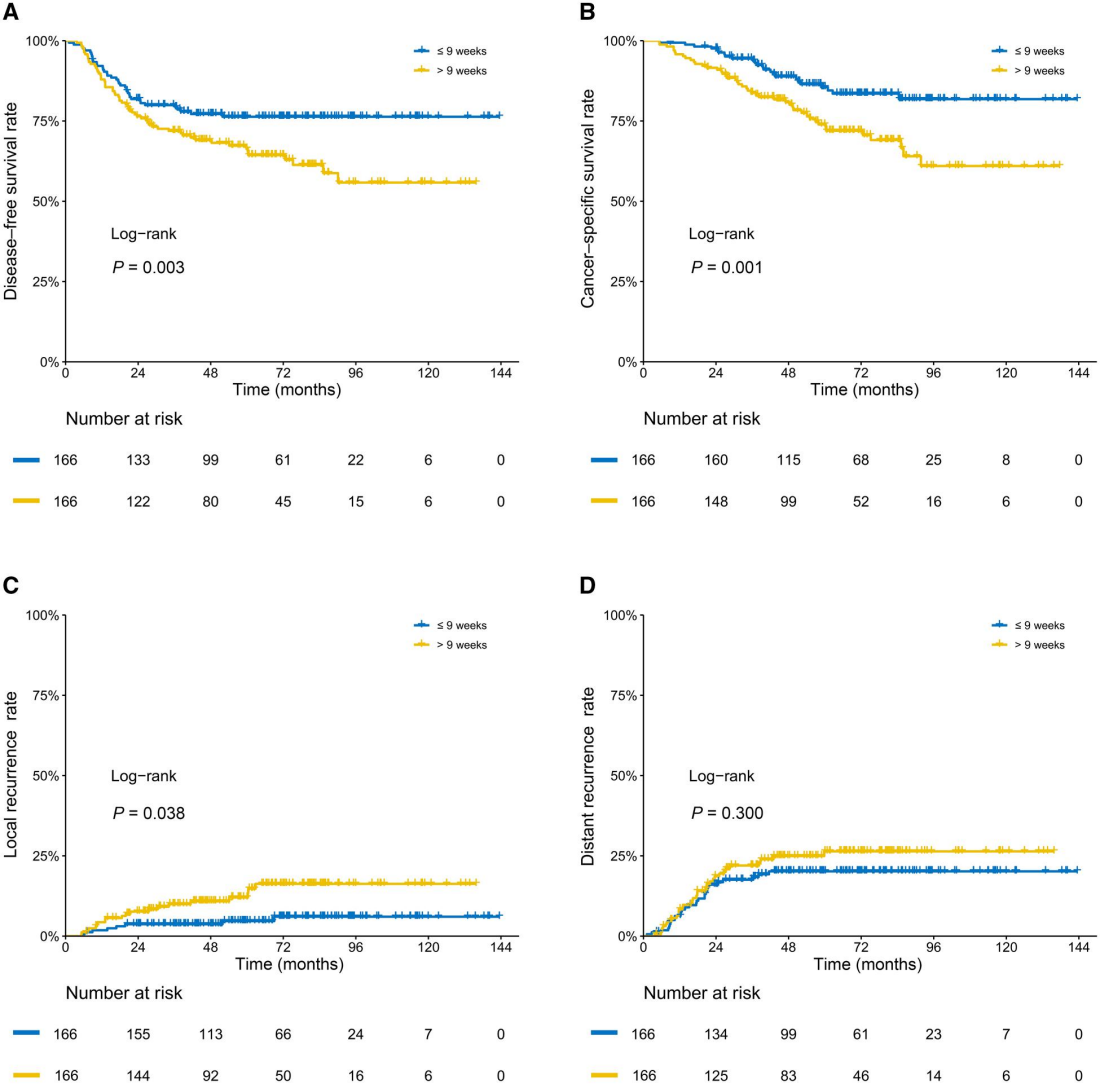
Kaplan–Meier estimates of (A) DFS, (B) CSS and cumulative incidence curves of (C) local recurrence and (D) distant recurrence in patients with a poor response to chemoradiotherapy in LARC after PSM.

In PSM analysis, patients in the delayed surgery group had a lower DFS rate at 5 years than those in the early surgery group (63.0% [95% confidence interval {CI}, 55.8%–71.1%] vs 76.3% [95% CI, 70.0%–83.2%]) and at 10 years (53.0% [95% CI, 43.6%–64.4%] vs 76.3% [95% CI, 70.0%–83.2%]; *P *=* *0.003) (Figure 3A). For CSS, results showed lower rates at 5 years in the delayed surgery group than in the early surgery group (72.9% [95% CI, 65.9%–80.6%] vs 85.5% [95% CI, 79.9%–91.5%]) and at 10 years (60.1% [95% CI, 49.9%–72.5%] vs 81.8% [95% CI, 75.1%–85.1%]; *P *=* *0.001) (Figure 3B). Longer waiting times were significantly linked to higher local recurrence rates (11.3% [95% CI, 6.0%–16.4%] vs 4.6% [95% CI, 1.1%–7.9%] at 5 years; 13.9% [95% CI, 7.5%–19.7%] vs 6.5% [95% CI, 2.1%–10.8%] at 10 years; *P *=* *0.038) (Figure 3C). Restricted mean survival time analysis showed a life-expectancy difference of 4.5 months (95% CI, 0.3–8.8; *P *=* *0.036) for DFS and 4.2 months (95% CI, 1.4–7.0; *P *=* *0.004) for CSS at 5 years (Supplementary Figure S3). At 10 years, the LED was 16.7 months (95% CI, 6.4–26.9; *P *=* *0.001) for DFS and 15.1 months (95% CI, 6.5–23.7; *P *=* *0.001) for CSS.

The univariate analysis is presented in Supplementary Table S2. Multivariate analysis indicated that delayed surgery was associated with poorer DFS (HR = 1.65; 95% CI, 1.21–2.24; *P *=* *0.001) and CSS (HR = 1.61; 95% CI, 1.12–2.32; *P *=* *0.009) (Table 2). Additionally, the distance from the anal verge, ypT stage, and ypN stage were identified as independent risk factors for both DFS and CSS. Tumor deposit showed a significant association with DFS, while adjuvant chemotherapy was notably associated with CSS.

**Table 2.** Multivariate analysis for DFS and CSS

Variable	DFS		CSS	
	HR (95% CI)	*P*-value	HR (95% CI)	*P*-value
Distance from the anal verge, cm				
≤5	1 [Ref]		1 [Ref]	
>5	0.68 (0.50–0.92)	0.011	0.62 (0.43–0.91)	0.013
Neoadjuvant chemotherapy				
XELOX	1 [Ref]			
Capecitabine	0.75 (0.53–1.06)	0.107		
Interval				
≤9 weeks	1 [Ref]		1 [Ref]	
>9 weeks	1.65 (1.21–2.24)	0.001	1.61 (1.12–2.32)	0.009
Surgery procedure				
Anterior resection			1 [Ref]	
Abdominoperineal resection			1.43 (0.97–2.11)	0.073
Hartmann			2.31 (0.90–5.92)	0.082
ypT stage				
0–2	1 [Ref]		1 [Ref]	
3	2.32 (1.53–3.51)	<0.001	2.42 (1.47–3.98)	0.001
4	3.21 (1.78–5.78)	<0.001	4.29 (2.20–8.37)	<0.001
ypN stage				
0	1 [Ref]		1 [Ref]	
1	1.93 (1.34–2.77)	<0.001	1.73 (1.11–2.69)	0.015
2	1.90 (1.09–3.33)	0.024	2.48 (1.34–4.58)	0.004
Tumor deposit				
Absence	1 [Ref]		1 [Ref]	
Presence	1.60 (1.03–2.51)	0.038	1.51 (0.86–2.58)	0.130
Nerve invasion				
Absence	1 [Ref]			
Presence	1.31 (0.84–2.04)	0.238		
Adjuvant chemotherapy				
No			1 [Ref]	
Yes			0.63 (0.40–0.97)	0.035

### Subgroup analysis

We further analysed patients with a pathological stage of ypT2N0 or ypN+ and the results showed that delayed surgery was also associated with worse survival (Supplementary Figures S4–S6). We also investigated the impact of baseline and perioperative factors on treatment efficacy (Supplementary Figures S7 and S8). The results showed that the benefit of early surgery on DFS was observed in nearly all of the subgroups, and this was also evident in the analysis of CSS across all of the subgroups.

### Sensitivity analysis

We conducted a sensitivity analysis by reclassifying patients into poor and good tumor responses according to the TRG system (Supplementary Figures S9 and S10). A total of 438 patients were classified as TRG2–3, defined as poor tumor response. The results indicated that delayed surgery did not significantly affect survival outcomes, suggesting that the TRG system may not be suitable for defining tumor response in this study.

### Immunohistopathological analysis

We performed immunostaining for CD8^+^ T cell infiltration in tumors (Supplementary Table S3 and Supplementary Figures S11–S13). Using the minimum *P*-value method, we determined a cut-off value of 300 cells/mm^2^, categorizing CD8^+^ T cell density into high-density (> 300 cells/mm^2^) and low-density (≤ 300 cells/mm^2^) (Supplementary Figure S14). Notably, patients in the ≤ 9 weeks group had higher CD8^+^ T cell infiltration than those in the > 9 weeks group (*P *=* *0.017) (Supplementary Figure S15). The results showed that patients with high stromal CD8^+^ T cell infiltration had better DFS (*P *=* *0.039) (Supplementary Figure S16).

## Discussion

This study investigated the optimal timing for surgery in LARC patients who had a poor tumor response after nCRT and explored the impact of delayed surgery on survival outcomes. Our results indicated that the optimal timing for surgery in this subset of patients was within 9 weeks after the completion of nCRT and delayed surgery was associated with worse DFS and CSS.

In recent years, achieving a pCR in patients with LARC has gained significant interest due to its association with favorable survival outcomes [4, 5, 20, 21]. Patients achieving complete tumor response can consider a watch-and-wait strategy, which can preserve the rectum and improve the quality of life. Previous studies have shown that extending the interval between nCRT and surgery could improve tumor response [8–10]. As this concept has gained acceptance, the interval between nCRT and surgery has been extended in current practice.

Results of the GRECCAR-6 trial indicated that a prolonged interval did not improve the 3-year DFS or OS rates in LARC patients (7 vs 11 weeks) [22]. Similarly, a meta-analysis showed that the lengthening of the surgical interval (≤6 vs > 6 weeks) did not affect local recurrence, distance metastasis, or OS [10]. This raises the question of whether the survival benefit of patients achieving a satisfactory tumor response is masked by those with a poor response. Patients with a pCR or near pCR may have better outcomes; however, improvements in DFS or OS were not observed in the entire population. Consequently, some patients who undergo delayed surgery may have a worse prognosis and resistant tumors may become more resistant and aggressive.

To the best of our knowledge, this is the first study to establish a clear linear relationship between the surgical interval and DFS and CSS in patients with a poor response to nCRT by using restricted cubic spline curves on a continuous scale. Our findings suggest that the optimal timing for surgery in this subgroup is within 9 weeks following nCRT. Delaying surgery for >9 weeks in the pursuit of better tumor response leads to poorer survival outcomes for the majority of patients (∼80% in this study), who will not achieve the desired pathological response. In the PSM analysis, the delayed surgery group exhibited a higher LR and lower CSS rate, with a 10-year survival difference reaching 21.7%.

A longer interval may be more suitable for good responders when aiming for a complete tumor response. A prospective observational study with weekly MRI assessments revealed that the tumor size rapidly decreased at the beginning of nCRT (26%/week), slowed to 7%/week in the last 2 weeks of nCRT, and finally to 1.3%/week in the last 5 weeks before surgery [23]. This suggests that poor responders who undergo delayed surgery have difficulty in achieving pCR. This is consistent with the results of a previous study by Probst *et al.* [24]. This study included 17,255 rectal cancer patients and showed that the pCR rate slowly increased with intervals of >8 weeks, peaking at ∼10–11 weeks after nCRT; however, the final pCR rate remained at <20% [24]. Generally, a 9-week interval is sufficient to achieve significant tumor regression, particularly for patients who respond well to the treatment.

Another point against delaying surgery is that it may adversely affect surgical outcomes. Patients receiving pelvic radiation are more likely to develop pelvic fibrosis and an extended waiting time may exacerbate this condition. This will complicate the surgical procedure and increase the likelihood of complications, especially when laparoscopic techniques are used [25, 26]. Such findings were noted in the GRECCAR-6 trial, in which patients in the late surgery group had higher overall morbidity and poorer quality of mesorectal resection [27]. Although our study does not provide direct evidence that delaying surgery leads to adverse surgical outcomes, it should raise our concerns.

Ideally, we should be able to select patients at an early stage following nCRT. Unfortunately, effective methods for precise patient selection are currently lacking. In contemporary clinical practice, patient selection primarily relies on preoperative Tumor Node Metastasis staging (ycTNM), which has clear limitations. Previous studies have indicated that MRI-based tumor regression grading (mrTRG) can predict survival outcomes for both favorable and poor responders [28, 29]. However, the low concordance between mrTRG and pathological tumor regression grading (pTRG) suggests that mrTRG cannot replace pTRG [30]. Additionally, endoscopic mucosal lesion regression grading is also an alternative method [31]. Research has shown that positron emission tomography–computed tomography scans may assist in identifying tumor response and allow the tailored selection of CRT-surgery intervals [32]. In recent years, MRI-based predictive models have demonstrated good predictive performance for achieving (near) complete pathological response (ypT0–1N0) following CRT in rectal cancer [33]. Xiao *et al.* [34] constructed a predictive genotype signature for pathologic complete response (pCR) in LARC after CRT and showed favorable results. Furthermore, Feng *et al.* [35] developed an artificial intelligence radiopathology integrated model that effectively predicts pCR in LARC patients after chemoradiotherapy. Patients who do not respond well to neoadjuvant therapy should be reevaluated and available staging modalities should be employed to make early selections. If a favorable tumor response is not observed within a specific waiting period (at least before 9 weeks), patients should undergo radical surgery without delay.

Developing the optimal neoadjuvant regimens has always been a key focus in the treatment of patients with LARC. Previous studies have demonstrated that more aggressive neoadjuvant treatment regimens, such as the implementation of intensified chemotherapy during radiotherapy or the addition of chemotherapy in the interval following radiotherapy, can further enhance the pCR rate and improve survival [21, 36, 37]. Our data have also shown that adjuvant chemotherapy was associated with better CSS in patients who responded poorly to chemoradiotherapy. In this regard, these patients may benefit from more aggressive neoadjuvant treatment regimens by shifting chemotherapy into the neoadjuvant period, also known as the total nCRT (TNT) strategy [38, 39]. However, it is still unclear whether the strategies discussed in this study to avoid surgical delays are fully applicable to patients undergoing TNT regimens. Perhaps a more aggressive neoadjuvant regimen, while identifying patients with a poor response to treatment at an early stage after chemoradiation and performing surgery on them in a timely manner, would achieve more satisfactory treatment outcomes.

We further investigated factors that affect survival outcomes in relation to the timing of surgery. Variations in lymphocyte infiltration within the tumor may explain the observed discrepancy. Notably, T cells, particularly CD8^+^ T cells, within the tumor microenvironment are positively correlated with DFS and OS [14]. In our current study, we found that patients who underwent early surgery exhibited a higher density of intra-tumoral CD8^+^ T cells than patients who had delayed surgery. This phenomenon may be attributed to the fact that radiotherapy can lead to the depletion of immune cells in the tumor microenvironment, as T cells are sensitive to radiation, resulting in their reduction [40]. In addition, an extended interval before surgery may exacerbate tissue fibrosis, thereby impeding T-cell infiltration and contributing to increased T-cell exhaustion, which increases the risk of tumor recurrence. These findings suggest that early surgical intervention may enhance the immune response to tumors, ultimately improving post-operative survival.

This study has several limitations. First, its retrospective design may have introduced bias due to patient selection and the heterogeneity of clinical practice. Second, ∼40% of the study population received standard nCRT (capecitabine-CRT), while the other portion received intensified chemoradiotherapy (xelox-CRT), which differs from the currently recommended total nCRT. This issue needs to be addressed in future multicenter prospective trials. Third, data on several variables were partially missing, such as mesorectal fascia, extramural vascular invasion, and mismatch repair status. Fourth, the sample size for immunostaining was relatively small and may have led to unstable results. Due to the fact that the response to neoadjuvant therapy can lead to varying histopathological changes even within a single tumor, obtaining partial residual tumors from large surgical specimens may not have adequately reflected the differences between tumors. Tissue slices covering the entire tumor bed are necessary to quantify and compare the density of CD8^+^ T cells within the tumor microenvironment. Unfortunately, only 42 samples met the criteria, while the remaining samples were excluded due to small tumor tissue specimens or insufficient tissue quality.

## Conclusions

This study identified the optimal timing for surgery in patients with LARC who exhibit a poor response to nCRT. Our findings challenge the prevailing trend of overextending the interval to achieve the maximum pCR rate. It is highly recommended to incorporate MRI regression grading, clinical staging, or other evaluation indicators to assess tumor response prior to surgery (at least before 9 weeks). Poor responders should receive radical surgery in time, without a delay.

## Ethics approval

All procedures conducted in this study involving human participants and human materials were approved by the Ethics Committee of Sun Yat-sen University Cancer Center (approval number: SL-B2024-086–01). The study was conducted in accordance with the Declaration of Helsinki. This study has been registered in the Research Registry (registration ID: researchregistry10242).

## Supplementary Material

goaf060_Supplementary_Data

## Data Availability

All data and analyses are available upon reasonable request. Please contact the corresponding author.
