# Supplementary material for: Delayed surgery for more than 9 weeks induces worse survival outcomes in locally advanced rectal cancer patients with poor response to neoadjuvant chemoradiotherapy: a propensity score matched cohort study
Source: Gastroenterol Rep (Oxf). 2025 Jul 2;13:goaf060. doi: 10.1093/gastro/goaf060 (PMC12221869; doi:10.1093/gastro/goaf060)
Supplement: goaf060_Supplementary_Data [file goaf060_supplementary_data.zip › 2024-626 Supplemental appendix.pdf]

Supplemental appendix

Delayed surgery for more than 9 weeks induces worse survival outcomes in locally advanced rectal cancer patients with poor response to neoadjuvant chemoradiotherapy: a propensity score-matched cohort study

Appendix 1

Supplementary Table S1. Characteristics of neoadjuvant therapy, surgery and postoperative treatment (unmatched)

Variable	No.of patients (%)			*P* value
	Overall (*n* = 583)	≤9 weeks (*n* = 404)	>9 weeks (*n* = 179)	
Age, median (IQR), years	57.0	56.0 (47.8-63.3)	58.0 (50.0-65.0)	0.054
Sex				0.305
Male	373	264 (65.3)	109 (60.9)	
Female	210	140 (34.7)	70 (39.1)	
BMI, median (IQR), kg/m2	25.1	24.9 (19.4-29.9)	25.2 (20.1-30.6)	0.246
ECOG				0.324
0	412	280 (69.3)	132 (73.7)	
1	171	124 (30.7)	47 (26.3)	
Comorbidity				0.213
0	470	320 (79.2)	150 (83.8)	
≥ 1	113	84 (20.8)	29 (16.2)	
CEA, median (IQR), ng/mL	3.8	3.7 (2.1-7.5)	3.9 (2.3-8.8)	0.321
Distance from the anal verge, median (IQR), cm	6.0	5.0 (4.0-8.0)	6.0 (4.0-8.0)	0.249
Tumor location				0.186
Anterior	198	147 (36.4)	51 (28.5)	
Lateral	53	39 (9.7)	14 (7.8)	
Posterior	49	32 (7.9)	17 (9.5)	
Circumferential	283	186 (46.0)	97 (54.2)	
Depth of tumor invasion, median (IQR), mm	15.0	15.0 (11.0-19.2)	15.4 (11.4-19.0)	0.887
Longitudinal length of tumor, median (IQR), mm	48.0	47.0 (36.0-58.0)	49.0 (38.0-60.0)	0.171
MRF				0.493
Positive	222	159 (39.4)	63 (35.2)	
Negative	324	218 (54.0)	106 (59.2)	
Unkown	37	27 (6.7)	10 (5.6)	
EMVI				0.979
Positive	214	149 (36.9)	65 (36.3)	
Negative	332	229 (56.7)	103 (57.5)	
Unkown	37	26 (6.4)	11 (6.2)	
cT stage				0.132
2	12	6 (1.5)	6 (3.4)	
3	354	254 (62.9)	100 (55.9)	
4	217	144 (35.6)	73 (40.8)	
cN stage				0.141
0	113	82 (20.3)	31 (17.3)	

1	307	219 (54.2)	88 (49.2)	
2	163	103 (25.5)	60 (33.5)	
Histological grade				0.449
Moderate, moderate-high, and high grades	460	322 (79.7)	138 (77.1)	
Low and low-moderate grades	90	57 (14.1)	33 (18.4)	
Signet-ring and mucinous adenocarcinoma	24	17 (4.2)	7 (3.9)	
Unknown	9	8 (2.0)	1 (0.6)	
Neoadjuvant chemotherapy				0.026
XELOX	368	267 (66.1)	101 (56.4)	
Capecitabine	215	137 (33.9)	78 (43.6)	
Cycles of XELOX				0.237
1	20	11 (2.7)	9 (5.0)	
2	101	78 (19.3)	23 (12.8)	
3	82	59 (14.6)	23 (12.8)	
4	165	119 (29.5)	46 (25.7)	
Grade 3/4 adverse events of chemotherapy*				
Any events	89	60 (14.9)	29 (16.2)	0.708
Anemia	42	27 (6.7)	15 (8.4)	0.489
Leukopenia	31	22 (5.4)	9 (5.0)	>0.999
Thrombocytopenia	23	15 (3.7)	8 (4.5)	0.650
Nausea/vomiting	8	5 (1.2)	3 (1.7)	0.706
Diarrhea	13	8 (2.0)	5 (2.8)	0.551
Liver injury	2	2 (0.5)	0	>0.999
Renal injury	3	2 (0.5)	1 (0.6)	>0.999
Neurotoxicity	4	3 (0.7)	1 (0.6)	>0.999
Surgical type				0.392
Laparoscopic	388	264 (65.3)	124 (69.3)	
Open	195	140 (34.7)	55 (30.7)	
Surgery procedure				0.433
Anterior resection	422	286 (70.8)	136 (76.0)	
Abdominoperineal resection	150	110 (27.2)	40 (22.4)	
Hartmann	11	8 (2.0)	3 (1.7)	
Temporary or permanent stoma				0.069
Yes	249	183 (45.3)	66 (36.9)	
No	334	221 (54.7)	113 (63.1)	
Status of Surgery				0.510
R0	553	386 (95.5)	167 (93.3)	
R1	22	13 (3.2)	9 (5.0)	
R2	8	5 (1.2)	3 (1.7)	
ypT stage				0.493
0/1	12	9 (2.2)	3 (1.7)	

2	151	101 (25.0)	50 (27.9)	
3	373	265 (65.6)	108 (60.3)	
4	47	29 (7.2)	18 (10.1)	
ypN stage				0.626
0	312	283 (70.1)	129 (72.1)	
1	140	101 (25.0)	39 (21.8)	
2	31	20 (5.0)	11 (6.2)	
Number of lymph nodes harvested after surgery, medium (IQR)	7.0	7.0 (4.0-8.0)	8.0 (5.0-9.0)	0.383
Tumor deposit				0.535
Presence	53	39 (9.7)	14 (7.8)	
Absence	530	365 (90.3)	165 (92.2)	
Nerve invasion				0.881
Presence	58	41 (10.1)	17 (9.5)	
Absence	525	363 (89.9)	162 (90.5)	
Vessel carcinoma embolus				0.289
Presence	40	31 (7.7)	9 (5.0)	
Absence	543	373 (92.3)	170 (95.0)	
MMR status				0.664
dMMR	25	18 (4.5)	7 (3.9)	
pMMR	399	263 (65.1)	136 (76.0)	
Unkown	159	123 (30.4)	36 (20.1)	
Surgical complications				
Any complication	64	43 (10.6)	21 (11.7)	0.774
Anastomotic leakage	27	16 (4.0)	11 (6.2)	0.345
Anastomotic bleeding	18	11 (1.9)	7 (1.7)	0.744
Abdominal infection	7	5 (1.2)	2 (1.1)	>0.999
Bowel obstruction	9	5 (1.2)	4 (2.2)	0.467
Wound infection	2	2 (0.5)	0	>0.999
Bleeding	3	2 (0.5)	1 (0.6)	>0.999
Dysuria	5	4 (1.0)	1 (0.6)	>0.999
Lymphatic leakage	4	3 (0.7)	1 (0.6)	>0.999
Clavien Dindo grade				>0.999
I	30	21 (5.2)	9 (5.0)	
II	25	16 (4.0)	9 (5.0)	
IIIa	6	4 (1.0)	2 (1.1)	
IIIb	3	2 (0.5)	1 (0.6)	
Adjuvant chemotherapy				0.019
No	97	57 (14.1)	40 (22.3)	
Yes	486	347 (85.9)	139 (77.7)	
Cycles of adjuvant chemotherapy				0.004
0	97	57 (14.1)	40 (22.3)	
1-3	127	84 (20.8)	43 (24.0)	

4-6	279	211 (52.2)	68 (38.0)	
Unknow	80	52 (12.9)	28 (15.6)	
Year of diagnosis				<0.001
2010-2013	186 (31.9)	150 (37.1)	36 (20.1)	
2014-2016	232 (39.8)	164 (40.6)	69 (38.5)	
2017-2020	164 (28.1)	90 (22.3)	74 (41.3)	

*National Cancer Institute Common Terminology Criteria for Adverse Events version 4.0.

Abbreviation: IQR, interquartile range; BMI, body mass index; ECOG, eastern cooperative oncology group; MRF, mesorectal fascia; EMVI, extramural vascular invasion; CEA, carcinoembryonic antigen; MMR, mismatch repair; dMMR, mismatch repair deficient; pMMR, mismatch repair proficient.

Appendix 2. Survival analysis before propensity score matching

Without considering the confounding factors between the early surgery (≤9 week) and delayed surgery (>9 week) groups, we examined the survival outcomes of both patient cohorts. As shown in the figure below (**Supplementary Figure S1**), delayed surgery was significantly associated with worse disease-free survival and cancer-specific survival,

We further divided the patients into 3 groups according to the interval time, as shown in figure below (**Supplementary Figure S2**). This part of the results showed that the survival rate tended to decrease with the extension of waiting time.

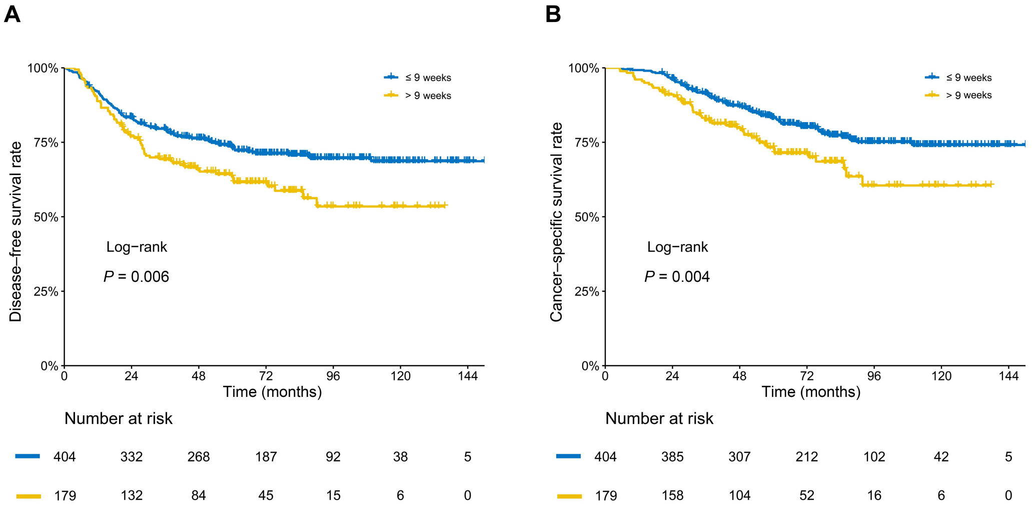

Supplementary Figure S1. Survival curve before PSM.

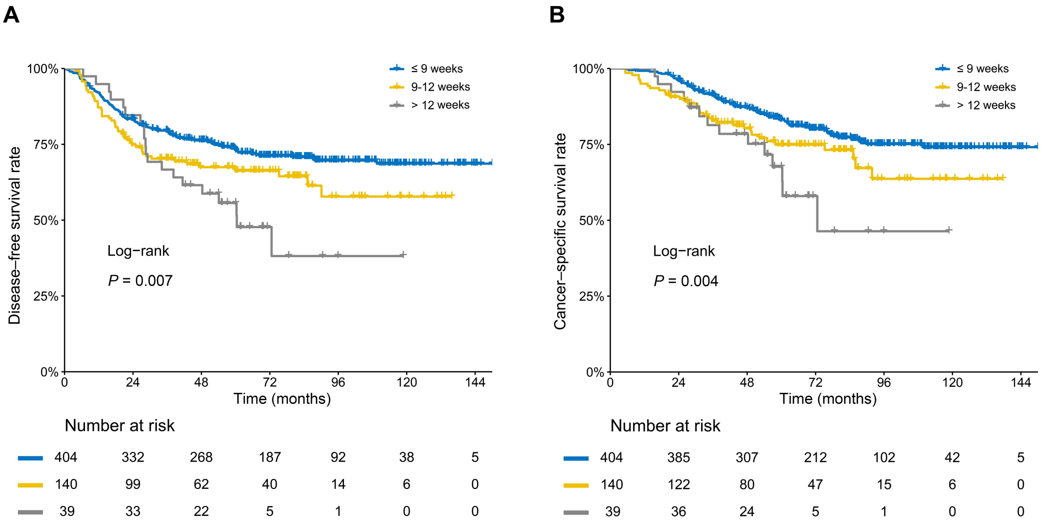

Supplementary Figure S2. Survival curve in patients stratified to 3 groups.

Appendix 3. Restricted mean survival time (RMST) analysis

RMST represents the area under the Kaplan-Meier curve. A and B represent the RMST of disease-free survival and cancer-specific survival at 5 years and life expectancy difference (LED) between the two groups was 4.5 months for DFS and 4.2 months for CSS. C and D represent the RMST of disease-free survival and cancer-specific survival at 10 years and life expectancy difference (LED) between the two groups was 16.7 months for DFS and 15.1 months for CSS.

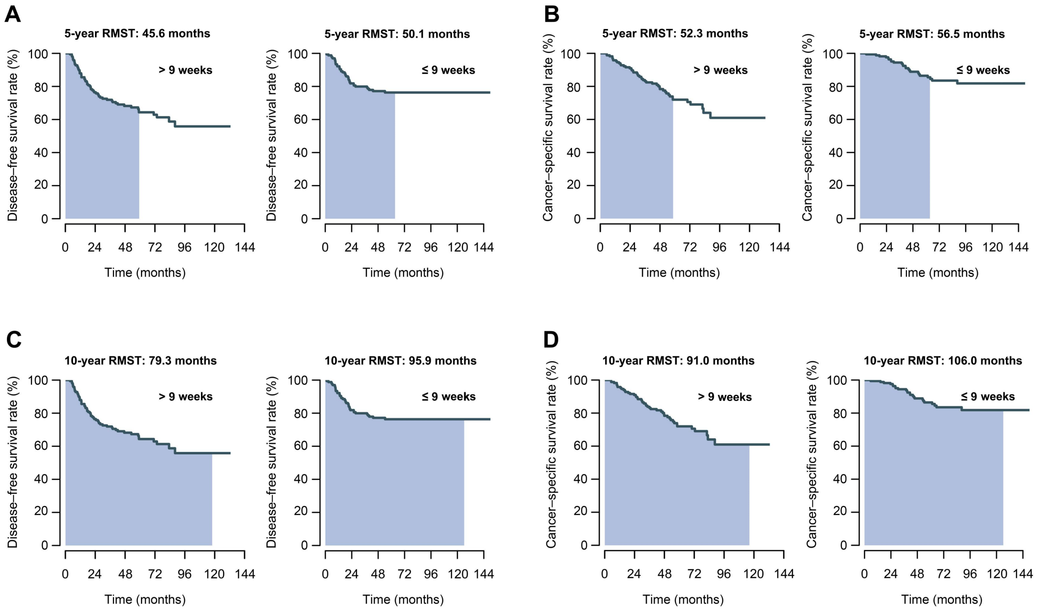

Supplementary Figure S3. Restricted mean survival time analysis at 5- and 10-years for two groups.

Appendix 4

Supplementary Table S2. Univariate analysis for disease-free survival and cancer-specific survival (*n* = 583)

Variable	DFS		CSS	
	HR (95% CI)	*P* value	HR (95% CI)	*P* value
Age, years	1.00 (0.98-1.01)	0.543	1.00 (0.99-1.02)	0.572
Sex				
Male	1 [Reference]		1 [Ref]	
Female	0.82 (0.60-1.12)	0.223	0.87 (0.61-1.25)	0.450
BMI, kg/m^2	1.00 (0.98-1.03)	0.695	1.01 (0.98-1.04)	0.568
ECOG				
0	1 [Ref]		1 [Ref]	
1	1.52 (1.12-2.05)	0.006	1.36 (0.95-1.94)	0.090
Comorbidity				
0	1 [Ref]		1 [Ref]	
≥ 1	0.86 (0.59-1.24)	0.416	0.76 (0.48-1.19)	0.230
CEA, ng/mL				
≤ 5	1 [Ref]		1 [Ref]	
> 5	1.22 (0.90-1.63)	0.197	1.12 (0.79-1.59)	0.527
Distance from the anal verge, cm				
≤ 5	1 [Ref]		1 [Ref]	
> 5	0.73 (0.55-0.98)	0.038	0.62 (0.44-0.88)	0.007
Tumor location				
Anterior	1 [Ref]		1 [Ref]	
Lateral	1.05 (0.61-1.82)	0.860	0.70 (0.32-1.56)	0.389
Posterior	1.02 (0.57-1.81)	0.959	1.05 (0.51-2.14)	0.900
Circumferential	1.03 (0.75-1.43)	0.842	1.25 (0.86-1.81)	0.240
Depth of tumor invasion, mm				
≤ 15	1 [Ref]		1 [Ref]	
16-30	0.94 (0.66-1.35)	0.745	1.10 (0.73-1.65)	0.662
> 30	1.43 (0.79-2.58)	0.238	1.38 (0.68-2.80)	0.374
Longitudinal length of tumor, mm				
≤ 50	1 [Ref]		1 [Ref]	
> 50	1.09 (0.80-1.50)	0.583	1.03 (0.71-1.50)	0.859
MRF				
Negative	1 [Ref]		1 [Ref]	
Positive	1.02 (0.57-1.81)	0.860	1.05 (0.51-2.14)	0.512
EMVI				
Negative	1 [Ref]		1 [Ref]	
Positive	1.01 (0.75-1.38)	0.931	1.06 (0.74-1.52)	0.740

cT stage				
2	1 [Ref]		1 [Ref]	
3	1.02 (0.37-2.76)	0.974	0.89 (0.28-2.82)	0.841
4	0.98 (0.36-2.70)	0.976	1.03 (0.32-3.30)	0.959
cN stage				
0	1 [Ref]		1 [Ref]	
1	0.74 (0.52-1.06)	0.098	0.81 (0.53-1.24)	0.332
2	0.70 (0.47-1.07)	0.098	0.78 (0.48-1.27)	0.324
Histological grade				
Moderate, moderate-high, and high grades	1 [Ref]		1 [Ref]	
Low and low-moderate grades	1.15 (0.78-1.71)	0.484	1.36 (0.87-2.12)	0.176
Signet-ring and mucinous adenocarcinoma	1.19 (0.58-2.42)	0.638	1.20 (0.53-2.74)	0.663
Neoadjuvant chemotherapy				
XELOX	1 [Ref]		1 [Ref]	
Capecitabine	0.62 (0.45-0.86)	0.005	0.69 (0.46-1.01)	0.057
Interval				
≤ 9 weeks	1 [Ref]		1 [Ref]	
> 9 weeks	1.52 (1.13-2.06)	0.006	1.67 (1.17-2.39)	0.004
Surgical type				
Laparoscopic	1 [Ref]		1 [Ref]	
Open	1.22 (0.90-1.65)	0.199	1.09 (0.76-1.57)	0.628
Surgery procedure				
Anterior resection	1 [Ref]		1 [Ref]	
Abdominoperineal resection	1.24 (0.90-1.71)	0.182	1.60 (1.12-2.30)	0.011
Hartmann	1.73 (0.71-4.24)	0.227	2.80 (1.14-6.91)	0.025
Temporary or permanent stoma				
Yes	1 [Ref]		1 [Ref]	
No	1.06 (0.79-1.42)	0.701	1.24 (0.88-1.75)	0.228
ypT stage				
0-2	1 [Ref]		1 [Ref]	
3	2.24 (1.49-3.37)	<0.001	2.21 (1.35-3.61)	0.002
4	3.15 (1.79-5.55)	<0.001	3.76 (1.95-7.24)	<0.001
ypN stage				
0	1 [Ref]		1 [Ref]	
1	2.33 (1.71-3.17)	<0.001	1.94 (1.34-2.81)	<0.001
2	2.38 (1.38-4.09)	0.002	2.57 (1.43-4.64)	0.002
MMR status				
pMMR	1 [Ref]		1 [Ref]	
dMMR	1.06 (0.49-2.27)	0.884	1.05 (0.43-2.60)	0.913

Postoperative lymph node retrieval				
< 12	1 [Ref]		1 [Ref]	
≥ 12	0.87 (0.61-1.24)	0.439	0.91 (0.60-1.37)	0.639
Tumor deposit				
Presence	1 [Ref]		1 [Ref]	
Absence	2.57 (1.76-3.77)	<0.001	2.24 (1.43-3.51)	<0.001
Nerve invasion				
Presence	1 [Ref]		1 [Ref]	
Absence	1.58 (1.03-2.41)	0.035	1.46 (0.89-2.40)	0.138
Vessel carcinoma embolus				
Presence	1 [Ref]		1 [Ref]	
Absence	1.03 (0.57-1.85)	0.788	0.84 (0.39-1.79)	0.645
Surgical complications				
Yes	1 [Ref]		1 [Ref]	
No	0.95 (0.59-1.53)		0.78 (0.43-1.42)	0.417
Adjuvant chemotherapy				
No	1 [Ref]		1 [Ref]	
Yes	0.88 (0.61-1.29)	0.519	0.65 (0.43-0.98)	0.041

Abbreviation: BMI, body mass index; ECOG, eastern cooperative oncology group; MRF, mesorectal fascia; EMVI, extramural vascular invasion; CEA, carcinoembryonic antigen; MMR, mismatch repair; dMMR, mismatch repair deficient; pMMR, mismatch repair proficient.

Appendix 5. Subgroup analysis

Patients with ypT2N0M0 typically have a good prognosis and do not require additional adjuvant chemotherapy. To further assess the rationale for classifying ypT2N0M0 patients as those with poor tumor response for analysis, we conducted a separate examination of this patient group to gain deeper insights into the impact of delayed surgery on the survival outcomes of those patients. A total of 117 patients had pathological staging ypT2N0M0, of whom 78 underwent surgery ≤9 weeks and 39 patients experienced a surgical delay >9 weeks. We performed survival analysis on this group of patients, as shown in the figure below (**Figure S4**). Although the difference was not statistically significant, the delayed surgery group showed a trend toward lower disease-free survival (DFS) compared with the early surgery group.

We found that there were differences in the year of diagnosis between the two groups. Therefore, we further drew the adjusted survival curve after adjusting for confounding factors, as shown in the figure below (**Supplementary Figure S5**). It can be seen that the DFS of the delayed surgery group is indeed lower than that of the early surgery group. These findings indicate that delayed surgery may still be associated with poor survival outcomes in patients with pathological stage ypT2N0M0.

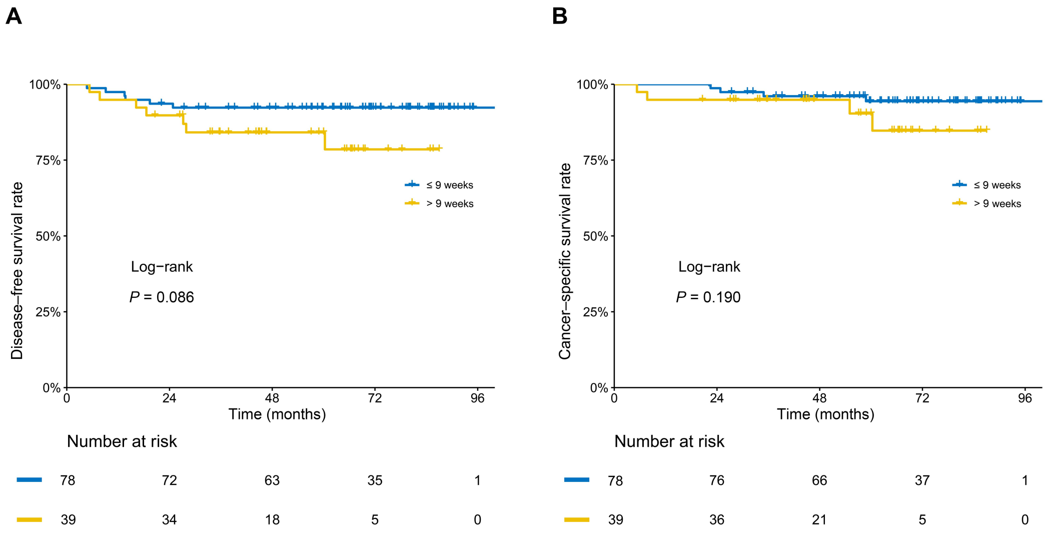

Supplementary Figure S4. Disease-free survival and cancer-specific survival of patients with ypT2N0M0.

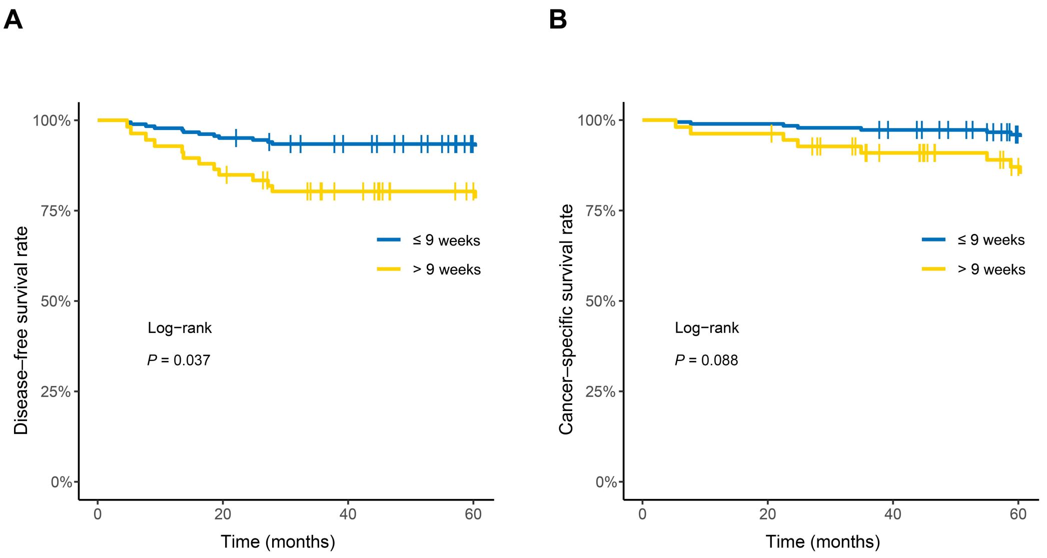

Supplementary Figure S5. Disease-free survival and cancer-specific survival of patients with ypT2N0M0 after adjusting the year of diagnosis (≤9 weeks vs >9 weeks).

Pathological lymph node positivity is a significant adverse prognostic factor. We have observed that some patients demonstrate remarkable regression of the primary tumor, despite persistent positivity in the lymph nodes. To further investigate the impact of delayed surgery on survival in patients with lymph node positivity, we conducted a separate survival analysis on a cohort of patients with a pathological staging of ypTxN+M0 (n = 171). Among the 171 patients, 121 underwent surgery ≤9 weeks, while 50 patients experienced a surgical delay >9 weeks. The findings showed that in this subgroup, the delayed surgery group had decreased disease-free survival and cancer-specific survival compared with the early surgery group (**Supplementary Figure S6**).

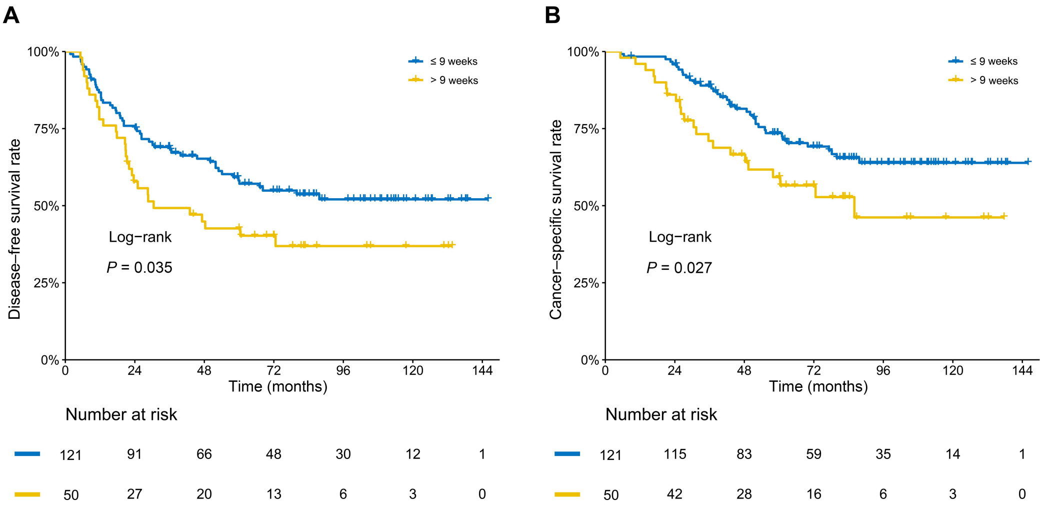

Supplementary Figure S6. Disease-free survival and cancer-specific survival of patients with ypTxN+M0 (≤9 weeks vs >9 weeks).

We additionally conducted subgroup analysis to assess the influence of baseline and perioperative factors on treatment effectiveness, as depicted below (**Supplementary Figure S7** and **S8**). In all subgroup analyses of variables, adjustments were made for the year of diagnosis to enhance result reliability.

The black dots represent hazard ratios (HR), horizontal lines indicate 95% confidence intervals, and the vertical dashed line signifies an HR of 1. Notably, a hazard ratio greater than 1, reflected by black dots positioned to the right, indicates a tendency favoring the treatment strategy of early surgery.

Subgroup	≤ 9 weeks (%)	> 9 weeks (%)	HR (95% CI)	*P* value	*P* for interaction
All patients	404 (100)	179 (100)	1.58 (1.15−2.16)	0.005	
Age, years					0.650
≤ 55	195 (48)	74 (41)	1.74 (1.09−2.78)	0.019	
> 55	209 (52)	105 (59)	1.52 (0.99−2.35)	0.058	
Sex					0.492
Male	264 (65)	109 (61)	1.46 (0.99−2.16)	0.054	
Female	140 (35)	70 (39)	2.07 (1.17−3.67)	0.013	
CEA, ng/mL					0.553
≤ 5	253 (63)	109 (61)	1.58 (1.04−2.40)	0.033	
> 5	149 (37)	70 (39)	1.49 (0.91−2.43)	0.110	
Distance from the anal verge, cm					0.980
≤ 5	207 (51)	81 (45)	1.53 (1.00−2.35)	0.051	
> 5	197 (49)	98 (55)	1.61 (1.00−2.59)	0.049	
cT stage					0.726
2−3	260 (64)	106 (59)	1.55 (1.04−2.30)	0.031	
4	144 (36)	73 (41)	1.79 (1.06−3.04)	0.030	
cN stage					0.308
0	82 (20)	31 (17)	1.62 (0.82−3.17)	0.162	
1	219 (54)	88 (49)	1.38 (0.87−2.18)	0.173	
2	103 (25)	60 (34)	2.42 (1.28−4.58)	0.007	
Degree of differentiation					0.771
Well	322 (81)	138 (78)	1.55 (1.09−2.21)	0.016	
Poor	74 (19)	40 (22)	1.33 (0.65−2.73)	0.434	
Neoadjuvant chemotherapy					0.752
Capecitabine + oxaliplatin	267 (66)	102 (57)	1.53 (1.04−2.25)	0.030	
Capecitabine	137 (34)	77 (43)	1.64 (0.91−2.93)	0.098	
Surgical procedure					0.380
Dixon	286 (71)	136 (76)	1.44 (0.98−2.12)	0.062	
Miles and Hartmann	118 (29)	43 (24)	2.02 (1.17−3.51)	0.012	
ypT stage					0.133
0−2	110 (27)	53 (30)	3.13 (1.43−6.82)	0.004	
3	265 (66)	108 (60)	1.51 (1.04−2.18)	0.031	
4	29 (7.2)	18 (10)	0.96 (0.30−3.06)	0.948	
ypN stage					0.463
0	283 (70)	129 (72)	1.57 (1.03−2.40)	0.035	
1	101 (25)	39 (22)	1.94 (1.14−3.30)	0.015	
2	20 (5.0)	11 (6.1)	0.81 (0.20−3.33)	0.766	
MMR status					0.101
pMMR	263 (94)	136 (95)	1.75 (1.17−2.61)	0.006	
dMMR	18 (6.4)	7 (4.9)	0.34 (0.07−1.79)	0.205	
Adjuvant chemotherapy					0.854
No	57 (14)	40 (22)	1.22 (0.62−2.39)	0.558	
Yes	347 (86)	139 (78)	1.52 (1.05−2.19)	0.027	

Adjusted for Year−of−diagnosis

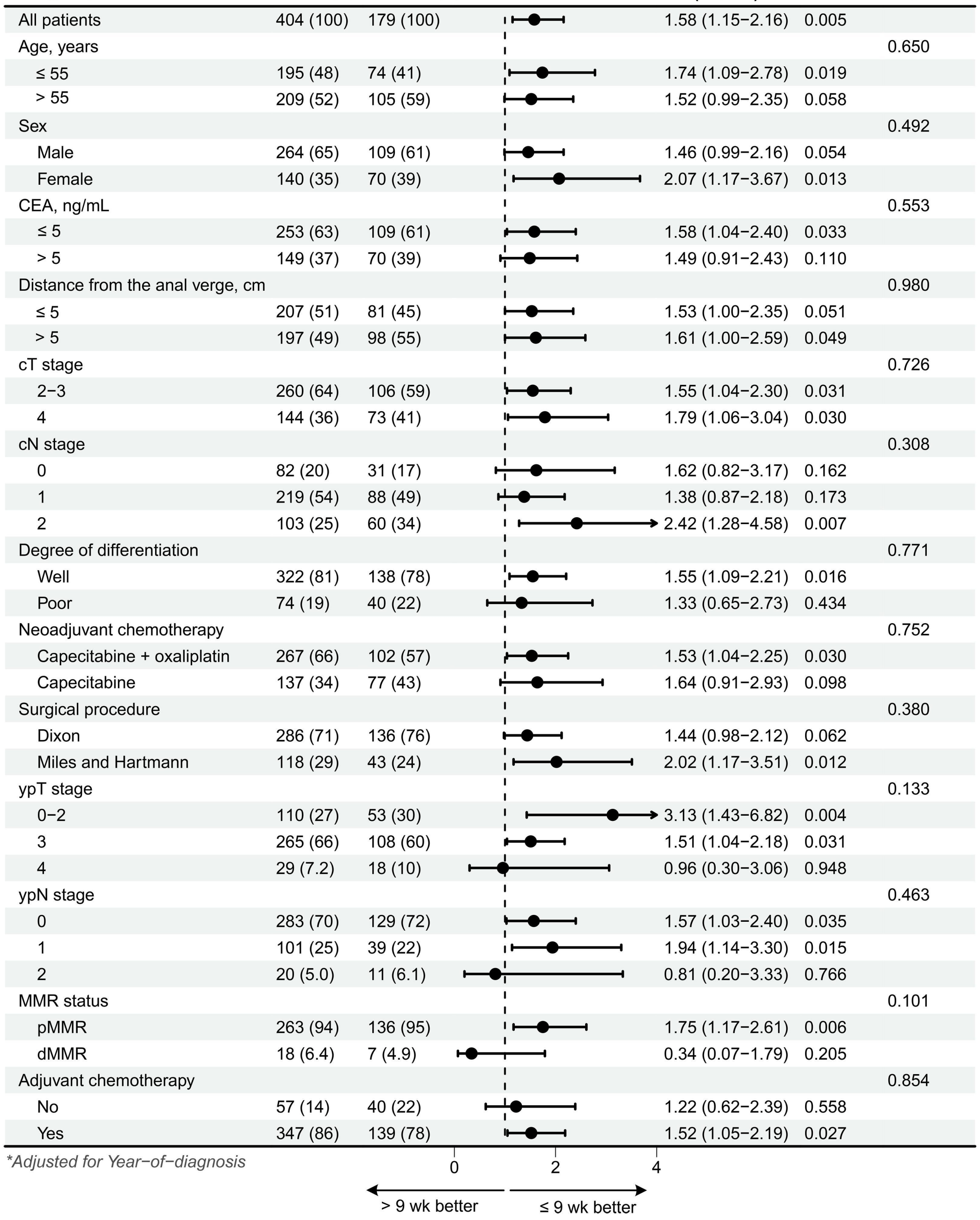

Supplementary Figure S7. Subgroup analysis of disease-free survival (*n* = 583).

Subgroup	≤ 9 weeks (%)	> 9 weeks (%)	HR (95% CI)	*P* value	*P* for interaction
All patients	404 (100)	179 (100)	1.85 (1.29−2.64)	0.001	
Age, years					0.831
≤ 55	195 (48)	74 (41)	2.01 (1.16−3.48)	0.012	
> 55	209 (52)	105 (59)	1.78 (1.10−2.87)	0.018	
Sex					0.269
Male	264 (65)	109 (61)	1.68 (1.08−2.62)	0.021	
Female	140 (35)	70 (39)	2.61 (1.37−4.96)	0.003	
CEA, ng/mL					0.888
≤ 5	253 (63)	109 (61)	2.02 (1.27−3.19)	0.003	
> 5	149 (37)	70 (39)	1.59 (0.89−2.84)	0.114	
Distance from the anal verge, cm					0.855
≤ 5	207 (51)	81 (45)	1.78 (1.11−2.87)	0.017	
> 5	197 (49)	98 (55)	1.87 (1.08−3.26)	0.027	
cT stage					0.880
2−3	260 (64)	106 (59)	1.92 (1.21−3.05)	0.006	
4	144 (36)	73 (41)	1.78 (1.00−3.18)	0.051	
cN stage					0.284
0	82 (20)	31 (17)	1.56 (0.72−3.36)	0.256	
1	219 (54)	88 (49)	1.83 (1.09−3.09)	0.023	
2	103 (25)	60 (34)	2.89 (1.36−6.14)	0.006	
Degree of differentiation					0.660
Well	322 (81)	138 (78)	1.72 (1.14−2.59)	0.010	
Poor	74 (19)	40 (22)	1.57 (0.71−3.44)	0.263	
Neoadjuvant chemotherapy					0.922
Capecitabine + oxaliplatin	267 (66)	102 (57)	1.90 (1.24−2.92)	0.003	
Capecitabine	137 (34)	77 (43)	1.62 (0.83−3.16)	0.155	
Surgical procedure					0.857
Dixon	286 (71)	136 (76)	1.98 (1.27−3.11)	0.003	
Miles and Hartmann	118 (29)	43 (24)	1.73 (0.95−3.14)	0.072	
ypT stage					0.081
0−2	110 (27)	53 (30)	5.58 (2.17−14.35)	<0.001	
3	265 (66)	108 (60)	1.66 (1.09−2.54)	0.019	
4	29 (7.2)	18 (10)	2.83 (0.66−12.16)	0.162	
ypN stage					0.819
0	283 (70)	129 (72)	1.73 (1.08−2.78)	0.023	
1	101 (25)	39 (22)	2.33 (1.23−4.40)	0.009	
2	20 (5.0)	11 (6.1)	1.75 (0.37−8.37)	0.481	
MMR status					0.457
pMMR	263 (94)	136 (95)	1.81 (1.14−2.88)	0.012	
dMMR	18 (6.4)	7 (4.9)	2.04 (0.35−11.69)	0.425	
Adjuvant chemotherapy					0.952
No	57 (14)	40 (22)	1.32 (0.65−2.68)	0.438	
Yes	347 (86)	139 (78)	1.73 (1.13−2.67)	0.012	

**Adjusted for Year−of−diagnosis*

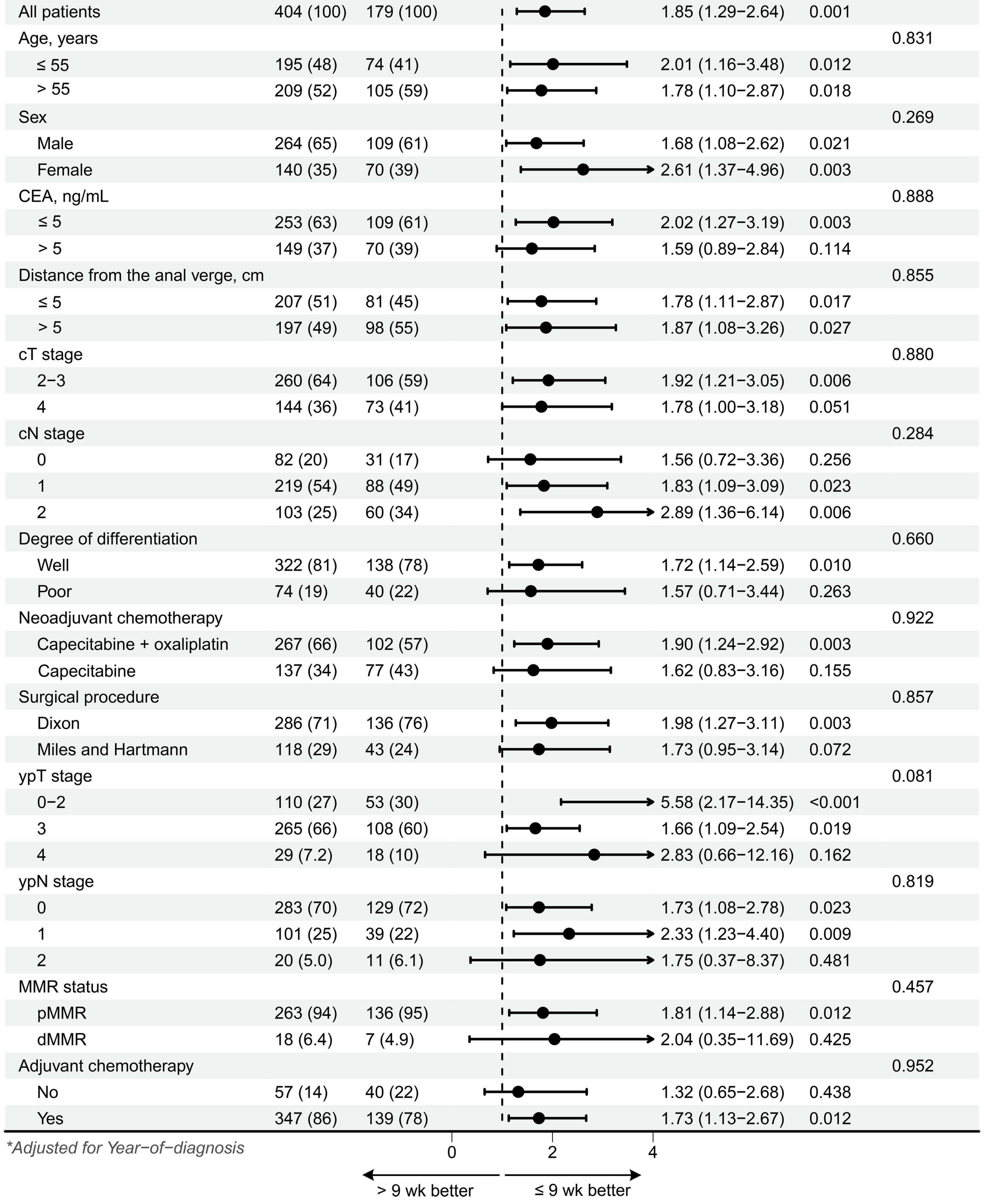

Supplementary Figure S8. Subgroup analysis of cancer-specific survival (*n* = 583).

Appendix 6. Sensitivity analysis

Another method of assessing tumor response involves employing the tumor regression grading (TRG) system. Several TRG systems have been developed to categorize tumor pathological response to nCRT[1, 2]. However, there remains no consensus regarding whether TRG serves as a prognostic factor for LARC[3-5].

The NCCN guidelines specify the most commonly used TRG grading system, which is a 4-point scale ranging from 0 to 3. TRG0: complete regression with no residual cancer cells; TRG1: almost complete regression with only one single residual cancer cell or a cluster of cancer cells; TRG2: moderate regression with many residual cancer cells; TRG3: minimal regression with nearly no cancer cells killed. TRG scoring is typically evaluated in the pathological report. Patients with TRG 2 to 3 were generally considered poor responders.

Among the 1,011 patients retrospectively analyzed in this study, 250 patients who did not meet the inclusion criteria were excluded. The relationship between the pathological staging system and TRG grading system of the remaining 761 patients was shown in the figure below (**Supplementary Figure S9**).

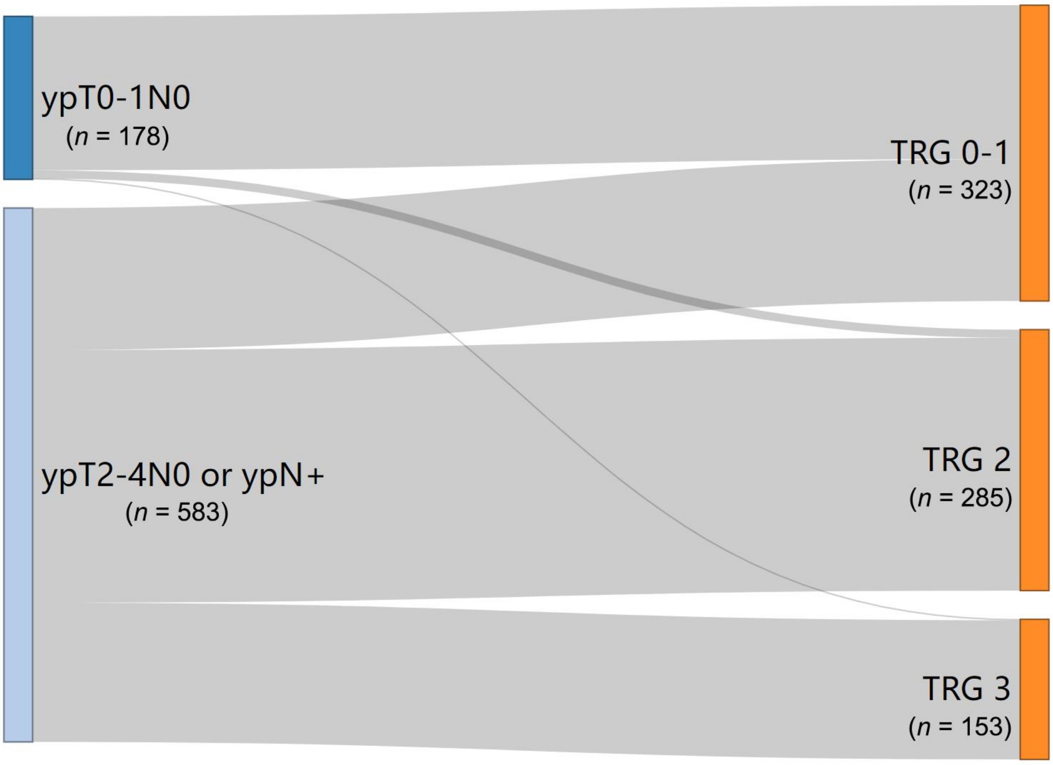

Supplementary Figure S9. The relationship between pathological stage and TRG grade in 761 patients.

Among the 438 patients categorized as TRG2 and 3, additional survival analysis was carried out (**Supplementary Figure S10**). Apparently, in the subgroup of patients classified as having poor tumor response according to the TRG grading system, there was no significant difference in survival outcomes between patients who underwent early surgery and those who underwent delayed surgery.

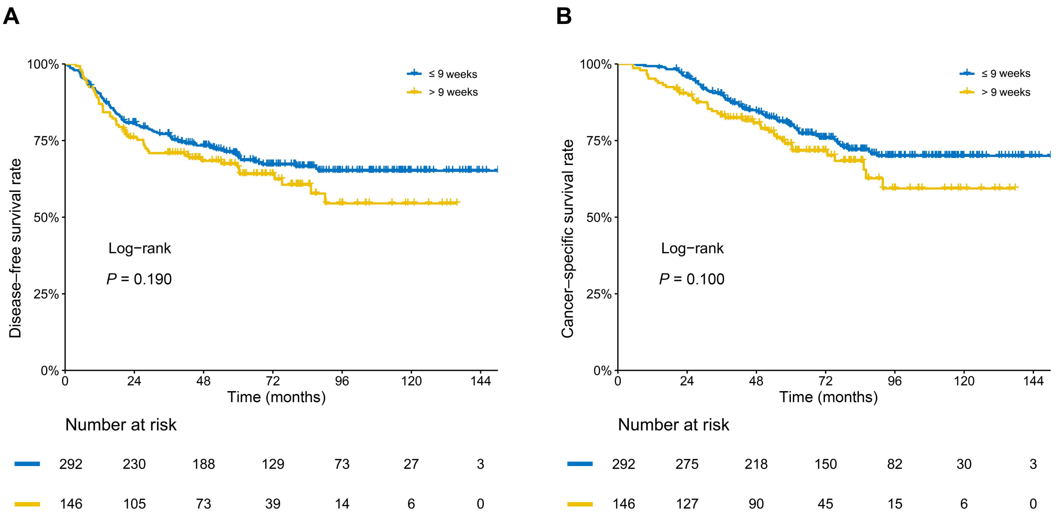

Supplementary Figure S10. Disease-free survival and cancer-specific survival of patients with TRG2 and 3 (≤9 weeks vs >9 weeks).

The reason for this may be that the TRG score predominantly assesses the regression of the primary tumor, neglecting to evaluate tumor involvement within lymph nodes. Consequently, certain patients may show significant regression of the primary tumor and be classified as TRG 0 to 1, despite still testing positive for lymph node involvement. These patients would also experience inferior survival outcomes due to the decision to delay surgery, as outlined above. Therefore, it may not be appropriate to use the TRG grading system to define tumor response in this study.

Appendix 7. Immunohistopathological analysis

This study involved the collection of surgical specimens from 42 patients diagnosed with locally advanced rectal cancer. These patients received neoadjuvant chemoradiotherapy and followed by surgery. Of these specimens, 20 belonged to the ≤9-week group, while 22 were from the >9-week group. The baseline characteristics of both groups are detailed in Table A6. Additionally, the following figure illustrates the distribution of the interval between neoadjuvant chemoradiotherapy and surgery among the 42 patients (**Supplementary Figure S11**).

Supplementary Table S3. Basic characteristics of patients

	**No. of patients (%)**		
**Variable**	**≤9 weeks (*n* = 20)**	**>9 weeks (*n* = 22)**	***P* value**
Age, median (IQR), years	51 (37-65)	55 (41-68)	0.772
Sex			0.913
Male	14 (70.0)	14 (63.6)	
Female	6 (30.0)	8 (36.4)	
CEA, median (IQR), ng/mL	4.0 (1.4-9.3)	6.9 (3.1-14.3)	0.371
Distance from the anal verge, cm			>0.999
≤ 5	10 (50.0)	12 (54.5)	
> 5	10 (50.0)	10 (45.5)	
cT stage			0.870
2	1 (5.0)	0	
3	7 (35.0)	8 (36.4)	
4	12 (60.0)	14 (63.6)	
cN stage			0.310
0	7 (35.0)	3 (13.6)	
1	10 (50.0)	14 (63.6)	
2	3 (15.0)	5 (22.7)	
Histological grade			0.344
Moderate, moderate-high, and high grades	14 (70.0)	18 (81.8)	
Low and low-moderate grades	2 (10.0)	3 (13.6)	
Signet-ring and mucinous adenocarcinoma	4 (20.0)	1 (4.5)	
Neoadjuvant chemotherapy			>0.999
XELOX	11 (55.0)	13 (59.1)	
Capecitabine	9 (45.0)	9 (40.1)	
Surgery procedure			>0.999
Anterior resection	15 (75.0)	16 (72.7)	
Abdominoperineal resection	5 (25.0)	6 (27.3)	
Hartmann	0	0	
ypT stage			0.538
0/1	0	0	

2	4 (20.0)	2 (9.1)	
3	15 (75.0)	18 (81.8)	
4	1 (5.0)	2 (9.1)	
ypN stage			0.104
0	16 (80.0)	12 (54.5)	
1	3 (15.0)	9 (40.9)	
2	1 (5.0)	1 (4.5)	
MMR status			0.283
dMMR	11 (55.0)	19 (86.4)	
pMMR	3 (15.0)	1 (4.5)	
Unkown	6 (30.0)	2 (9.1)	
Adjuvant chemotherapy			0.598
No	2 (10.0)	1 (4.5)	
Yes	18 (90.0)	21 (95.5)	

Abbreviation: IQR, interquartile range; CEA, carcinoembryonic antigen; MMR, mismatch repair; dMMR, mismatch repair deficient; pMMR, mismatch repair proficient.

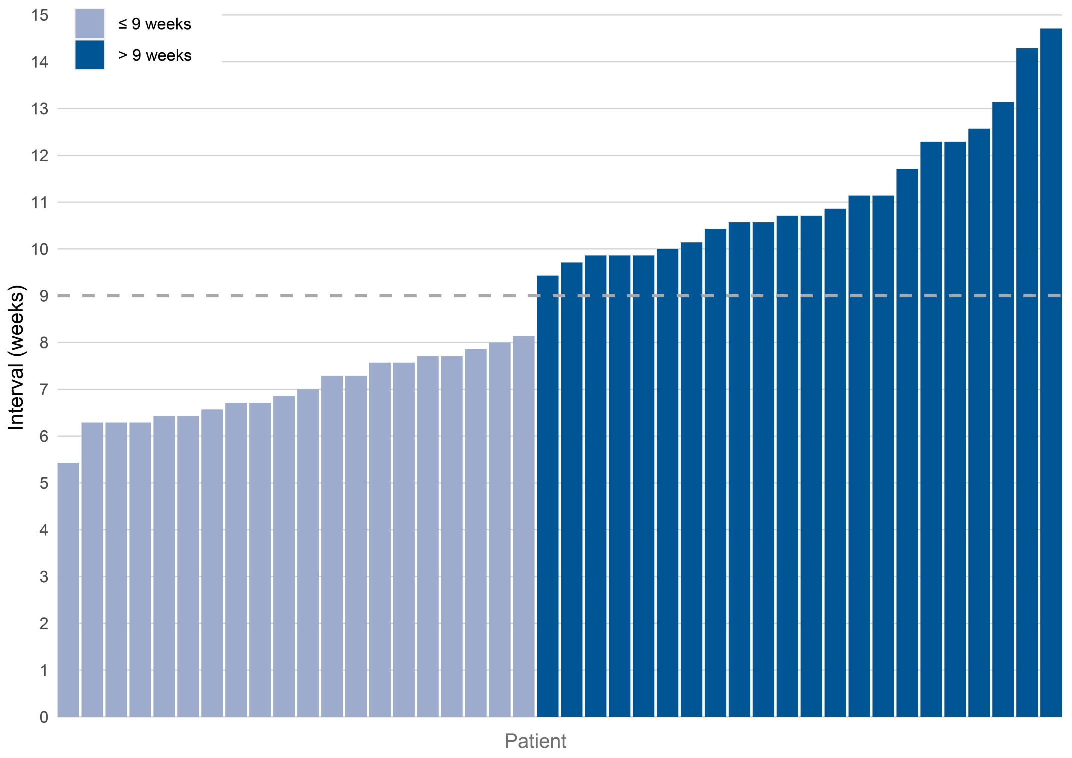

Supplementary Figure S11. The interval between neoadjuvant chemoradiotherapy and surgery in 42 patients.

The survival analysis results revealed that the survival of patients in the >9-week group was worse compared to that in the ≤9-week group (P = 0.011 and P = 0.009) (**Supplementary Figure S12**).

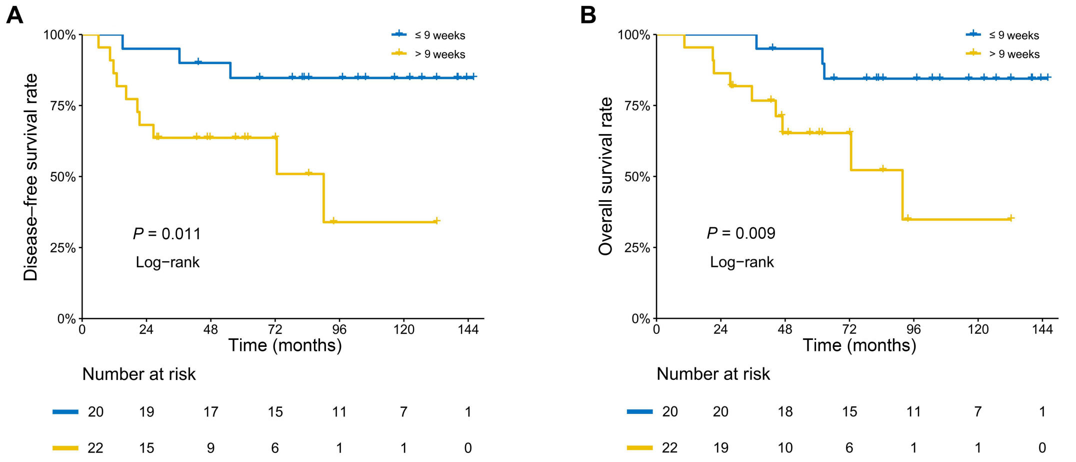

Supplementary Figure S12. Survival curves of disease-free survival and overall survival in 42 patients (≤9 weeks vs >9 weeks).

We sliced rectal cancer specimens following neoadjuvant therapy and subsequently stained the CD8^{+} T cells within the tumor, as illustrated in the figure below (**Supplementary Figure S13**). Notably, the density of CD8^{+} T cells in the early surgery group is significantly higher than that in the delayed surgery group.

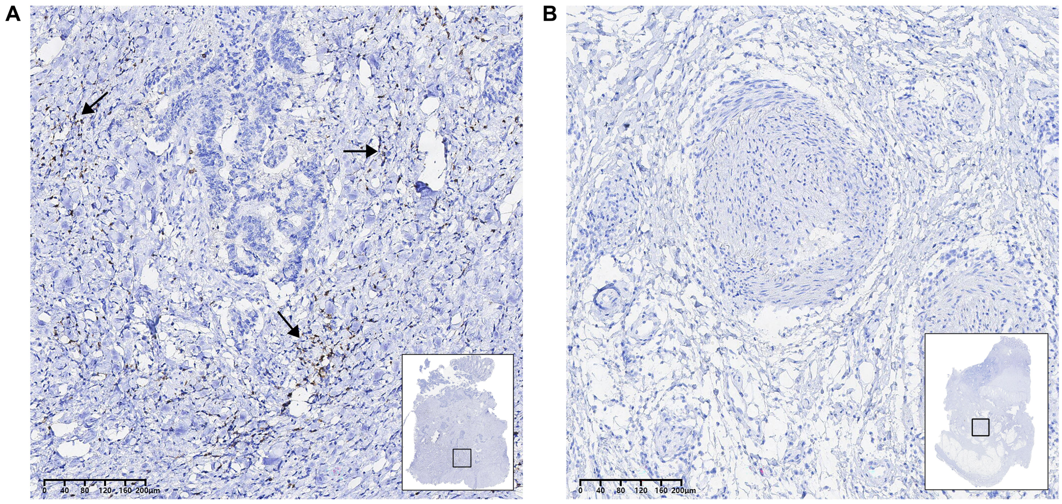

Supplementary Figure S13. Immunostaining of rectal cancer specimens. (A) CD8 staining in patients undergoing early surgery (≤9 weeks). The black arrow indicates CD8^{+} T cells in the immunostaining. (B) CD8 staining in patients undergoing delayed surgery (>9 weeks).

As mentioned in the main text, we categorized 42 patients into two groups based on the cutoff value of 300 cells/mm^2 for intra-tumoral $CD8^+$ T cell density: those with high density (> 300 cells/mm^2) and those with low density (≤ 300 cells/mm^2) (**Supplementary Figure S14**). The results showed that the density of $CD8^+$ T cells in the tumors of the early surgery group was higher than that of the delayed surgery group (**Supplementary Figure S15**). We found that patients with high $CD8^+$ T cell density had better disease-free survival compared to those with low density (P = 0.039) (**Supplementary Figure S16**).

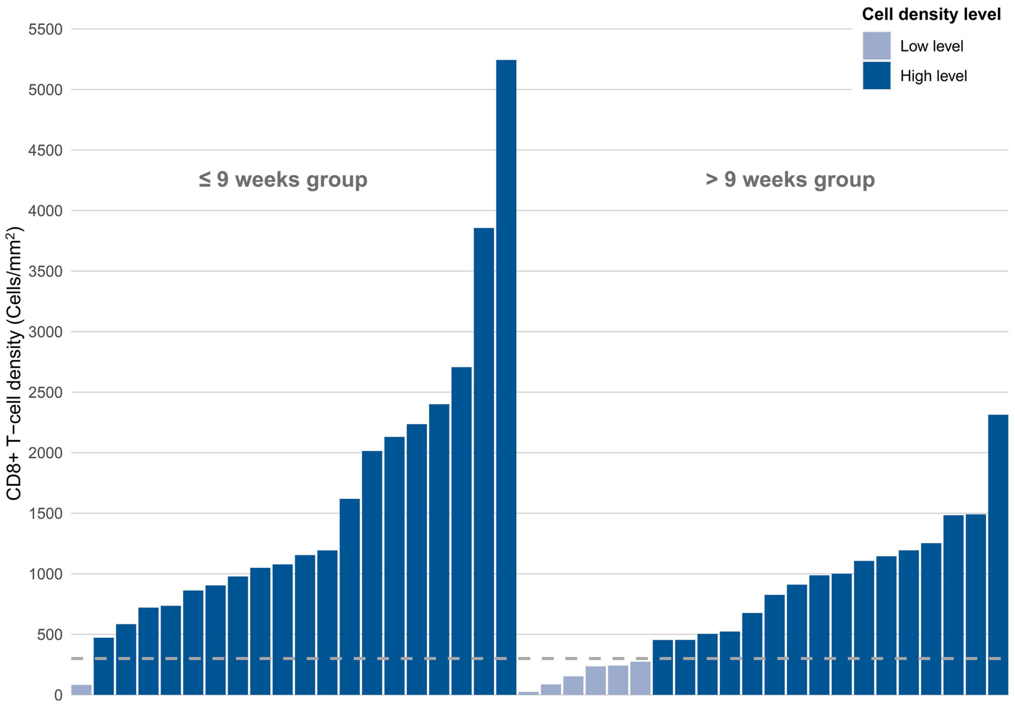

Supplementary Figure S14. Distribution of the stromal $CD8^+$ T cell density (cells/mm^2) between the early surgery group and the delayed surgery group. The dashed line indicates 300 cells/mm^2.

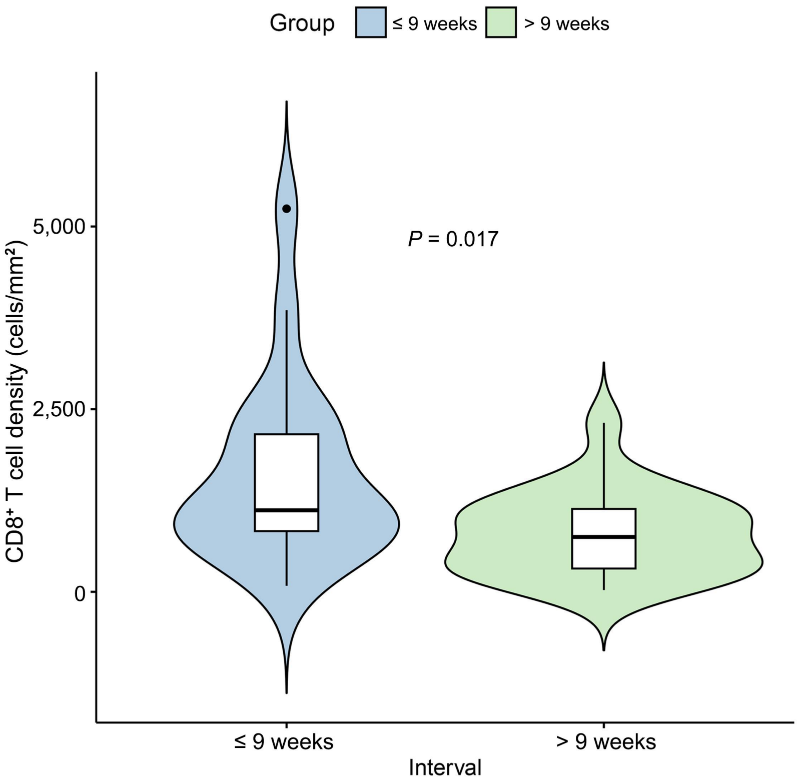

Supplementary Figure S15. Comparison of CD8^{+} T cell density (cells/mm^{2}) between the early surgery group and the delayed surgery group.

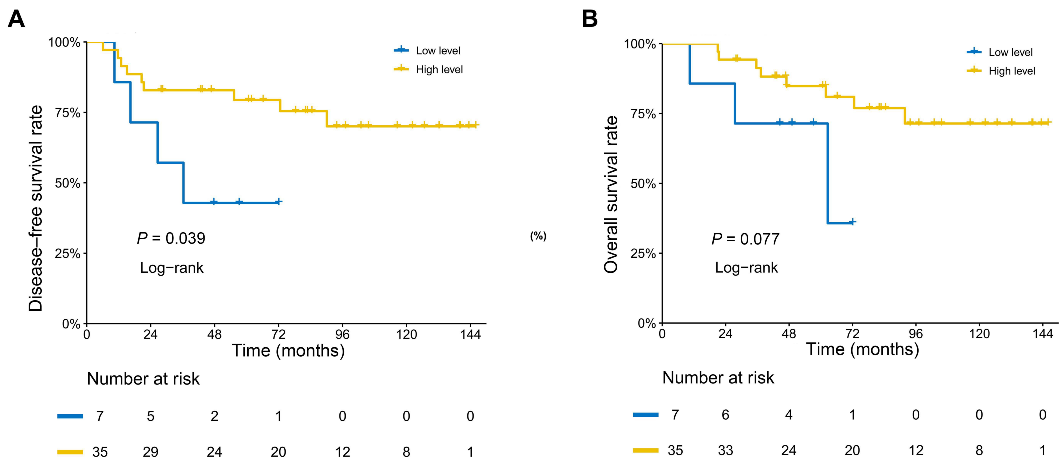

Supplementary Figure S16. Survival curves of disease-free survival and overall survival in patients with high or low intra-tumoral CD8^{+} T cell density.

References

1. Kim SH, Chang HJ, Kim DY, et al. What Is the Ideal Tumor Regression Grading System in Rectal Cancer Patients after Preoperative Chemoradiotherapy? *Cancer Res Treat.* Jul 2016;48(3):998-1009.

2. Germani P, Di Candido F, Léonard D, et al. Contemporary snapshot of tumor regression grade (TRG) distribution in locally advanced rectal cancer: a cross sectional multicentric experience. *Updates Surg.* Oct 2021;73(5):1795-1803.

3. Abdul-Jalil KI, Sheehan KM, Kehoe J, et al. The prognostic value of tumour regression grade following neoadjuvant chemoradiation therapy for rectal cancer. *Colorectal Dis.* Jan 2014;16(1):O16-25.

4. Fokas E, Ströbel P, Fietkau R, et al. Tumor Regression Grading After Preoperative Chemoradiotherapy as a Prognostic Factor and Individual-Level Surrogate for Disease-Free Survival in Rectal Cancer. *J Natl Cancer Inst.* Dec 1 2017;109(12).

5. Erlandsson J, Lörinc E, Ahlberg M, et al. Tumour regression after radiotherapy for rectal cancer - Results from the randomised Stockholm III trial. *Radiother Oncol.* Jun 2019;135:178-186.
